# Analysing potential data security losses in organisations based on subsequent users logins

**DOI:** 10.1371/journal.pone.0286856

**Published:** 2023-08-24

**Authors:** Benjamin Aziz

**Affiliations:** School of Computing, University of Portsmouth, Portsmouth, United Kingdom; Al-Balqa Applied University Prince Abdullah bin Ghazi Faculty of Information Technology, JORDAN

## Abstract

Multi-user computer environments pose potential threats to users data in organisations, in that unauthorised subsequent users who log on to the same computer could leak, alter or delete data belonging to users who previously logged in to the same computer. Such a threat is inspired by Locard’s exchange principle, which states (in its digital form) that every interaction with a system must ultimately leave some trace, and as a result, such trace could carry with it sensitive information that subsequent interactions may obtain without authorisation. Therefore, we attempt in this paper to define a subsequent users analysis that calculates this potential loss in data security based on data visibility and sensitivity values. We outline how such analysis can be used in the real world to enhance decision making process when logging in to a shared computer. We adopt a data-driven approach in defining our analysis and we demonstrate the validity of the analysis over a large open Cybersecurity dataset, which associates users with computers.

## Introduction

Security, in the context of computing and information systems, is a term often used to refer to the Confidentiality, Integrity and Availability (CIA) triad of properties [1]. The importance and criticality of security was recognised in computing environments in works as early as [2] and as soon as computing systems became a shared resource, thereby allowing users’ data, information and computations to be also shared. Despite all the technological advances that have been achieved over the years in terms of the protection of information and digital resources, using various methods such as cryptography, access control and context isolation mechanisms, security concerns seem to be still at the top of the issues facing modern digital infrastructures and systems. On the other hand, with the increasing availability of large and open datasets, new solutions to the problems of security are emerging, which rely more on data-driven approaches (e.g. [3–5]) as opposed to model-driven approaches (e.g. [6, 7]) or even adopting a purely engineering-based solution [8]. Data, in fact, are increasingly viewed as sources of information and knowledge that can increase the security and robustness of systems.

Nonetheless, there are still many factors that hinder the adoption of “open data” in this respect. Such factors are, above all, political, as a result of the continuing diffidence on the part of commercial organisations to share their data because of the fear that such data may eventually reveal sensitive information about those organisations and their security weaknesses. However, there are also technical factors, related to the consistency, quality and the lack of consensus on the nature of variables that should be monitored and the metrics that should be used to quantify the definition of security itself [9]. There are also philosophical questions related to whether past data are in any way relevant to future events [10]. Nonetheless, in recent years, this trend has started to shift with the arrival of large open datasets backed by the reliability and reputation of large organisations, e.g. Verizon’s VCDB [11], CERT’s Vulnerability Notes Database at Carnegie Mellon University [12], SecRepo [13], CAIDA [14] and LANL [15]. Moreover, we are starting to notice increasing usage of such datasets in the security and digital forensics research communities.

We consider in this paper the problem of security as a risk that arises from the sharing of computer resources among users. This risk remains high even in recent times; sharing computers without supervision among employees in an enterprise has been highlighted as one of the main causes of data leakage worldwide [16]. We define a subsequent users analysis that we use in defining the visibility data have when at rest on a computer. We consider that subsequent users who log on to a computer are essentially a threat to any sensitive data left behind by previous users on that computer, where such threat could lead to losses in the CIA data security properties. We quantify the potential for such losses as a product of the data visibility and sensitivity values.

Despite strong account isolation properties in modern computing paradigms (e.g. cloud computing), users may still leave data on shared storage space on computers (e.g. temporary or shared folders) or any other trace pointing to their presence on the system, which could be accessed and analysed by subsequent, and potentially unauthorised, users. Such behaviour could be due to negligence, and in general, it is assumed to obey Locard’s exchange principle, which states that users will always leave behind some data or trace in a digital environment scene [17]. Therefore, subsequent users will always pose a threat to information about past users.

In formalising the solution to whether we can calculate the potential data security loss, we adopt a data-driven research approach that refines the main question to a set of more detailed sub-questions, which are then expressed as a set of mathematical functions that can be calculated in terms of the adopted dataset. We apply this approach to an open Cyber security dataset called the “User-Computer Authentication Associations in Time” dataset [18, 19] made available by the Los Alamos National Laboratory in New Mexico. In this, we aim to demonstrate how datasets can be used to guide the definition and understanding of the scale of potential of data security losses in multi-user computing environments. Our approach is not new—in fact, the analysis of computer usage based on user-computer association datasets can be observed as a subject of research in literature from as early as [20], who demonstrated how groups of users could be identified from the examination of CPU processing time.

### Research gaps

The work presented in this paper aims to address a number of research gaps, which we summarise by the following bullet points:

- There is a gap represented by the shortage in literature of clear methodologies transforming research questions into a set of computable functions that can then be applied to dataset(s) from which answers (and therefore knowledge) are generated to satisfy the original research question.
- There is also clear under-utilisation in scientific research literature of open data, specially within the context of digital forensics, security and risk analysis. Most literature is focused on the classical machine-learning approach, which uses data as a means for validating the efficiency and accuracy of the machine learning algorithms themselves, without producing any new insights or knowledge into how such data can inform the forensics analysts or the risk and security research communities.
- Much of literature aims at separating method of security and digital forensics *analysis* from the concept of the *usability* of secure mechanisms that are part of the interface of a computing environment (e.g. access control mechanisms). There is rarely work that combines the two aspects, in order to provide a notion of how *risky* the computing environment is, for example, before a user logs on to the machine and starts using it.

### Contributions

The current paper contributes to the field of digital forensics and information security in the following three ways:

- We define a methodology, which breaks down a broad research question in terms of its most basic (concrete) parts of sub-questions, ending with the implementation of each such sub-question using a mathematical function. The application of the various functions identified to a dataset provides then a scientifically verifiable answer to the main question. This contribution addresses the first research gap above.
- The paper defines a precise expression of Locard’s exchange principle in terms of what we call the subsequent users analysis, and then demonstrates how the method above can be used to calculate the results of this analysis in an open dataset that associates users to computers in time. This contribution aims at addressing the second research gap above, but constructing a systematic *data-drive* approach to the problem of subsequent users interfering with a shared computing environment.
- The third and final contribution of the paper is to combine our exact expression of Locard’s exchange principle, which we term the subsequent users analysis, with an expression of data security called sensitivity, to yield a risk model that can be used as part of the interface to computers in a network. This interface becomes useful in informing users of the level of risk associated with leaking their sensitive data once they log on to some computer. This contribution addresses the *usability* question of such a model, which is highlighted by our third research gap above.

### Structure

The rest of the paper is structured as follows. In Section Background, we present some background on the topic of this paper. In Section Related Work, we discuss literature related to our analysis. In Section Methodology, we outline our data-driven research approach. In Section An Overview of the LANL UCAAT Dataset, we give an overview of the “User-Computer Authentication Associations in Time” dataset from the Los Alamos National Laboratory, which we use as the case study underlying our analysis. In Section Referencessec:analysis, we define a subsequent users analysis, which is then used to derive the overall average for the dataset. In Section Security Analysis and Usability, we use the subsequent users analysis to define measures for the loss of data security. Finally, we conclude the paper in Section Conclusion and discuss future research directions for this work.

## Background

Edmond Locard, the famous 19^th^ century French criminologist, remarked in his police investigation manual book [21, §III, p. 79]: “Il est impossible au malfaiteur d’agir, et surtout d’agir avec l’intensité que suppose l’action criminelle, sans laisser des traces de son passage [It is impossible for a criminal to act, and especially to act with the intensity that a criminal action requires, without leaving traces of their presence]”. This remark was later to be coined as the *Principle of Exchange* by Reginald Morrish [22]. Nowadays, this is commonly known as Locard’s exchange principle, which one can summarise as saying that no interference with a crime scene can be completed without leaving some trace. This principle has also been applied in the world of computer and digital forensics. In 1966, Milo Arthur Bennett [23] became the first person ever to be prosecuted for computer-related crime, when he used a newly installed computer programme at National City Bank of Minneapolis, to pay himself money that would cancel some debt he owed. Traces of his activities were discovered once the programme was brought offline due to a fault, and manual auditing of his accounts revealed that he had embezzled $1,357. The principle is accepted nowadays as a fundamental concept underlying all disciplines of digital forensics [24], where every interaction with a computing element is assumed to leave one kind of trace or another, even if that trace is as implicit as a record of the heat dissipated from the computing element as a result of its computations [25]. But the arrival of the new wave of *data-driven* paradigms rendered this principle even more important due to the abundant availability of digital datasets, which can be studied to learn new methods that intruders use to interfere with systems and commit digital crime. We are now able to test new theories on such datasets, and increase the knowledge associated with methods of Cyber crime. As a result, our approach here is data-driven as it ultimately applies and tests the theory using open data.

The idea that (sensitive) information may be leaked to unauthorised users through the sharing of computational and storage resources was first explicitly formulated by Butler Lampson in 1973 in his seminal paper “A note on the confinement problem” [26]. Lampson describes one of the aspects of this problem in [26, p.2] as follows:

“The service [a possibly untrustworthy programme] may write into a permanent file in its owner’s directory. The owner can then come around at his leisure and collect the data”.

The problem then arises if the computer or storage environment being used is multi-user or multi-tenancy (i.e. shared). This means that the data written by the untrustworthy programme (or service) become vulnerable to being accessed by subsequent (unauthorised) users, either unintentionally, or as a result of a malicious strategy. Later, Denning [27] formalised the notion of information flow as a desirable security property using a mathematical framework based on lattice structures.

Our work, at its most fundamental level, is inspired by Locard’s exchange principle, as one manifestation of Lampson’s confinement problem. We assume that a user who logs on to a computer will undoubtedly leave some trace behind (exchange principle), which may be of value to subsequent users logging in to the same computer (confinement problem). However, we take a risk-oriented approach to the evaluation of whether such trace does leak any meaningful sensitive information, to those subsequent users, or not, rather than adopting a policy enforcement approach (e.g. the enforcement of safe information flows). Our risk formulation then relies on both components; a quantification of the visibility of data on a computer as expressed through the *arrival* of subsequent users to the same computer, and a quantification of the security sensitivity of the data themselves (i.e. traces) left by previous users. Therefore, our control of the information flow becomes a user-driven decision, rather than an actual policy.

## Related work

One of the earliest works that addressed the problem of security in the context of multi-user programmes in computing environments was that of James Anderson [2], who defined the concept of a *reference monitor* as an isolation layer that separates and protects the trusted internal resources from the untrusted external world of user programs, applications and systems attempting to access those resources. This protection is achieved by allowing the reference monitor to evaluate whether an attempted to access a resource is an *authorised* attempt or not, by referring to a local set of rules called a *security policy*. The ideas presented by James Anderson in [2] led to the evolution of security in multi-user computing systems over time and the appearance of many solution paradigms that were based on a variety of approaches including access control matrix models [28], access control lists [29], multilevel security using information flow [27, 30, 31], public-key cryptography [32] and security based on cryptographic communication protocols [33]. To a large extent, such approaches were either model or engineering driven.

With the arrival of virtualisation techniques in the late 1990s, the problem of user data security became one of the central issues in what became to be known as *multi-tenancy environments* underlying models such as Cloud computing [34]. The sharing of resources became synonymous with cost reduction and resource provision on-demand [35]. However, many of the data security issues, such as data loss, modification and unauthorized access in Cloud Computing, remain even nowadays at the forefront of the concerns [36–39] when using Cloud computing, driven by existing vulnerabilities in the technologies underlying multi-tenancy platforms, in general. According to [40], such vulnerabilities can be summarised in terms of malicious insiders, insecure interfaces and shared technologies, which lead to high risks of data security losses. Another common cause is the lack of user control according to [37]. But, despite the strong isolation properties that multi-tenancy platforms claim to enforce, privacy leaks are still common [36].

Another area of applications where multi-user environments are dominant are collaborative systems [41], where security concerns have been highlighted in such systems in works such as [42–44]. Collaborative systems allow multiple user teams based in geographically dispersed locations to work together, supporting communication, coordination and cooperation among those teams. However, this also means that such teams of users may share computational storage space and resources, therefore, opening up the possibility of leaking sensitive data via such shared platforms.

Of strong relevance to our work here is that of [45, 46], who propose a mathematical framework for the calculation of the risk of data privacy loss in online social media networks. They define this risk as the product of two factors: the sensitivity level of the data resource intended to be shared over the network, and the number of unauthorised users who may have access to that resource. In this paper, we adopt a similar definition to the risk of losing data security, however, we base this on the data-driven approach and assume Locard’s exchange principle [17] as the main justification for data visibility on computers. There is also another indirect justification in that understanding numbers related to subsequent user associations to a computer also facilitates forensic analysis of similarities in the bahaviour of users (e.g. as was shown by [47]).

Other works have exploited the LANL Cyber security datasets for their case studies in order to either prove the validity of the method being introduced, or to simply understand the presence of any patterns in these datasets. For example, in [48], the authors analysed the NetFlow dataset (part of [49]) and discovered a number of interesting patterns, including the clustering of network connections into three main clusters with rare portals classified as having abnormal network connections that need to be investigated. In [50], the authors apply their newly defined attention-seeking analysis using recurrent neural networks to analyse the LANL datasets. The analysis is useful in a number of case studies presented in the paper, including word and character tokenisation, suggesting for example, that certain domains or characters are more popular than others. Similarly, the authors in [51] design a new algorithm for the detection of lateral movement, called LMTracker, which is applied to the LANL datasets in order to detect advanced persistent threats.

Literature is rich with works that tackle the question of “how” information may be accessed, the security mechanisms controlling such access and its impact on the security of the overall system or organisation. In this paper, we do not deal with such mechanisms, but rather focus on the measuring of the probability of unauthorised data leaks, given historical data about user access to computers. We also build here on our past experiences analysing the LANL datasets, e.g. [52], where we apply common quantitative methods, such as timeline, local averages and exponentially weighted moving average analysis to discover the presence of three anomalies in LANL’s DNS dataset, and in [53], where we use the UCAAT dataset again to formalise a data-driven model of Chinese Walls [26].

Some other works, such as [54, 55], have proposed a different approach to follow when considering the method of data-driven forensics; that is, to synthesize artificial datasets for the purpose of testing a specific digital forensics technique. The motivation for synthesizing a digital forensics dataset is that existing datasets “*quickly become outdated […] are often too academic, too specific, too synthetic […] and/or too anonymised.*” quoting [55]. Thus, a synthetic dataset may suit better a testing study.

Finally, we can say that our work also touches on the intersection of data science and digital forensics, as it is driven by one of forensics most fundamental ideas, Locard’s exchange principle. Horsman and Lyle [56] define this intersection as consisting of three main categories of datasets: tool and process evaluation datasets, actions datasets and scenario-based datasets. We position our work here in the second category concerned with the analysis of an actions (user logins) dataset. Our methodology itself, outlined in the next section, is an example of the *shift* required from the investigative and legal question, to the scientific and forensic question, as underlined by Inman and Rudin [57]. This shift is further described as “question creation” by Pollitt [58, §5] and further described as being part of the identification phase in a digital forensics process. Investigating the scientific question itself requires ultimately the availability of data. Grajeda et al. [59] provided a recent survey on the availability of open datasets for digital forensics research, and the conclusion was that such datasets still remain, unfortunately, scarce.

Table 1 highlights how works most related to ours compare, in terms of whether they cover the following criteria (which have all been covered by our work here):

R1 . Presence of an implementable methodology linking a security or digital forensics research question to a model that can be validated.

R2 . Presence of some model of security or digital forensics principle, e.g. Locard’s principle, defined and validated in a scientific manner, e.g. via application to a dataset.

R3 . Presence of a clear relationship between some security or digital forensics concept and risk, defined in a methodological manner.

R4 . Presence of a clear usability method or approach in which the presented work is made relatable to existing everyday usage scenarios in computing environments.

**Table 1:** A comparative table of related works.

Related Works	R1	R2	R3	R4
[56–58]	✓			
[20, 42–44, 54, 55, 59]		✓		
[40, 52]			✓	
[2, 37]				✓
[47, 49]		✓		✓
[53]			✓	✓
[45, 46]		✓	✓	✓
[50, 51]	✓	✓		✓

## Methodology

**Fig 1:**
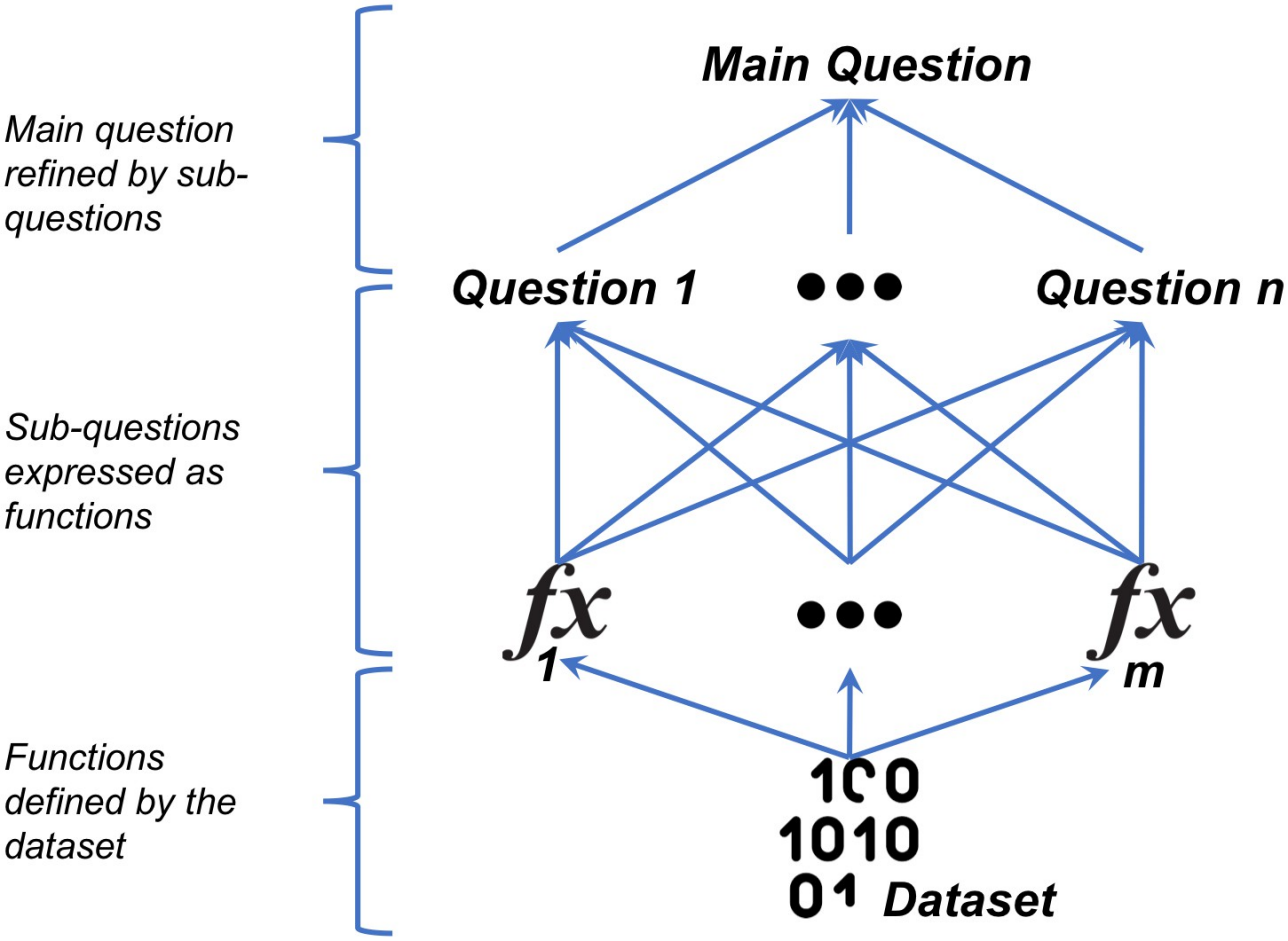
The data-driven research approach.

Our data-driven research approach can be summed up by the three main stages shown in Fig 1.

In the first stage of this approach, we break down the main research question into a set of smaller more refined sub-questions. In fact, this step could be iterative in that the sub-questions themselves could be refined further on until we are satisfied that level of detail in a question is close enough to its mathematical form. For example, in our case, the main question is this: *can we utilise the potential for the loss of data security in a multi-user computing environment?*

**Fig 2:**
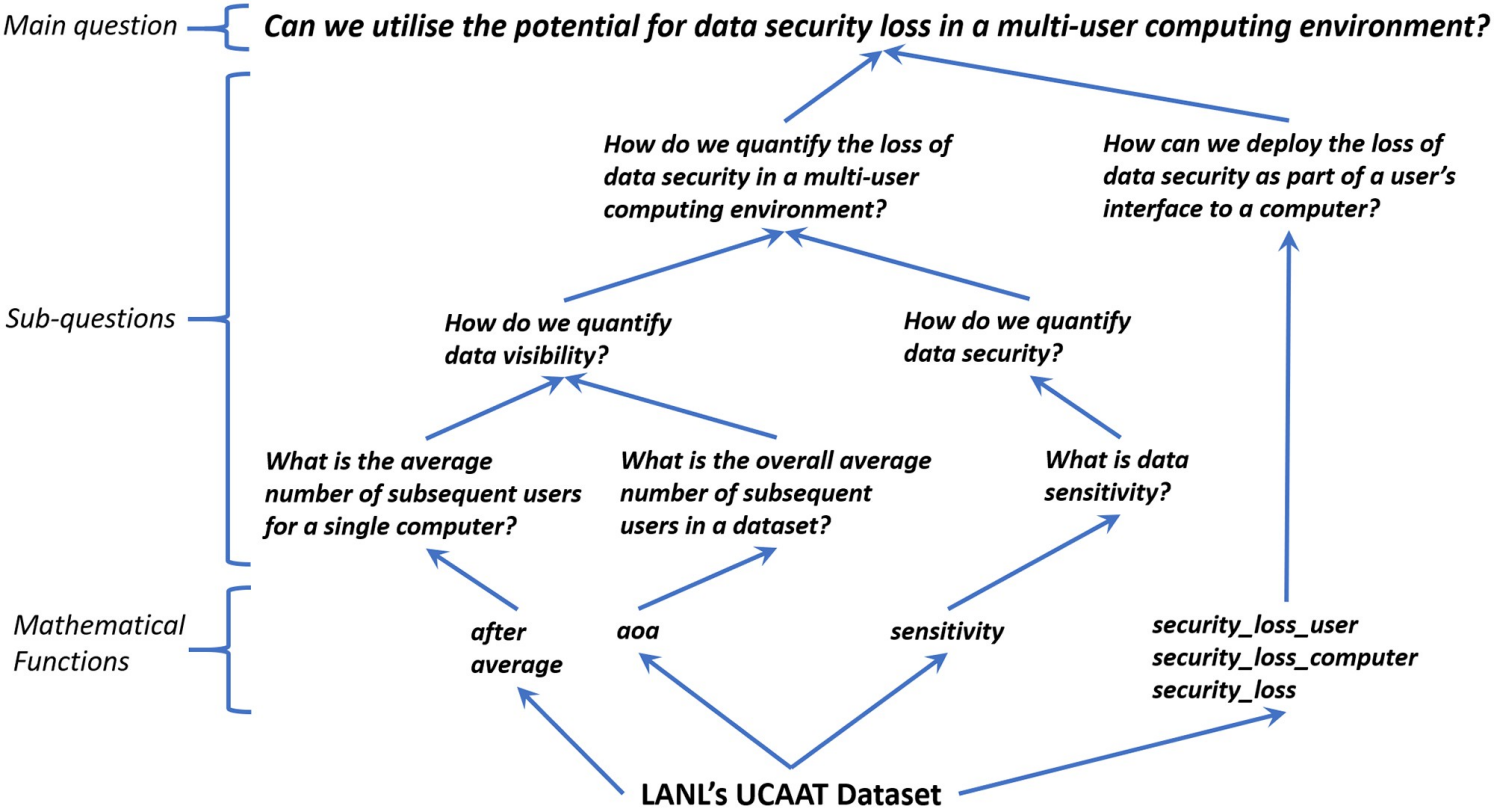
The data-driven research approach.

Once questions have been refined to a level that can be defined clearly, we express these questions in terms of a set of functions, which in practical terms, provide the answer to these questions and hence indirectly to the main research question. This is the second stage of the approach. Looking again at the example of our analysis later, we shall express the data visibility question in terms of the subsequent users functions (Section Subsequent Users Analysis) and the data sensitivity question in terms of the data sensitivity function (Section Security Analysis and Usability). In general, it is possible that a question is expressed in terms of multiple functions and similarly, a function expresses more than one question. Fig 2 illustrates how this question can be broken down into more concrete sub-questions, until finally we can implement the last layer of sub-questions using mathematical functions. The arrows point towards higher levels of abstraction.

What renders this approach “data-driven” is that the definitions of the basic functions are set-theoretic, i.e. are defined solely by the underlying dataset itself, in the third stage of the approach. For example, this is the case with the *after* function (Section Subsequent Users Analysis), which has a definition based on information extracted directly from the dataset itself. Naturally, there may also exist some functions, which will be higher-order in the sense that their definitions require other functions. Once all the functions have been defined then we have an answer to the main research question. For example, we shall define the potential for data security loss functions (Section Security Analysis and Usability), which answers our main research question, in terms of the data visibility and data sensitivity functions. We also demonstrate in Section Security Analysis and Usability how the question of usability of the subsequent users analysis is implemented.

We next give an overview of the dataset we used in our research in this paper.

## An overview of the LANL UCAAT dataset

The Los Alamos National Laboratory (LANL) Open Cyber Security Science [60] provides currently three open Cyber security datasets. These are the Unified Host and Network dataset [61], which is a collection of network and computer events, the Comprehensive, Multi-Source Cyber Security Events dataset [62], which is a collection of Cyber security event datasets collected from the internal network, and finally, the User-Computer Authentication Associations in Time (UCAAT) dataset [18, 19], which is a dataset representing successful authentications of users to computers. We focus in this paper on the third dataset as it contains the exact kind of data underlying the research question considered in this paper. We now give a brief overview of the UCAAT dataset. The dataset [18, 19] was collected by the Los Alamos National Laboratory (LANL) over a period of 9 months and represents 708,304,516 successful authentication events from users to computers. An example of some lines in the dataset is shown on Table 2.

**Table 2:** Example data lines from the UCAAT dataset.

Time	User	Computer
1	U1	C2
2	U2	C3
3	U3	C4
6	U4	C5
7	U4	C5
7	U5	C6
8	U6	C7
11	U7	C8
12	U8	C9

Each line contains three meta-data elements; the first represents the time at which the authentication event occurred, the second represents the user who logged in to the computer, and the third the computer on which the login happened. The time epoch starts at 1 with a resolution of 1 second. To enhance anonymity, the time frame of the actual data collection is not provided. There are in total 11,362 users, represented by the pseudo values for U*i* and 22,284 computers, represented by the pseudo values for C*j*, where $i\in N^{+}$ and $j\in N^{+}$ represent the number of the user and the computer, respectively. In the rest of the paper, we call the set of all users U and the set of all computers C. Since we are considering here the problem of user data security in multi-user environments, we only consider multi-user computers in UCAAT. There were 2808 such computers. We exclude the rest as being single-user (or personal) computers, for which our research question does not apply.

The dataset is available either as a single compressed file (size 2.3GB) or as a set of 9 individual files (with sizes ranging from 177MB to 273MB).

## Subsequent users analysis

**Fig 3:**
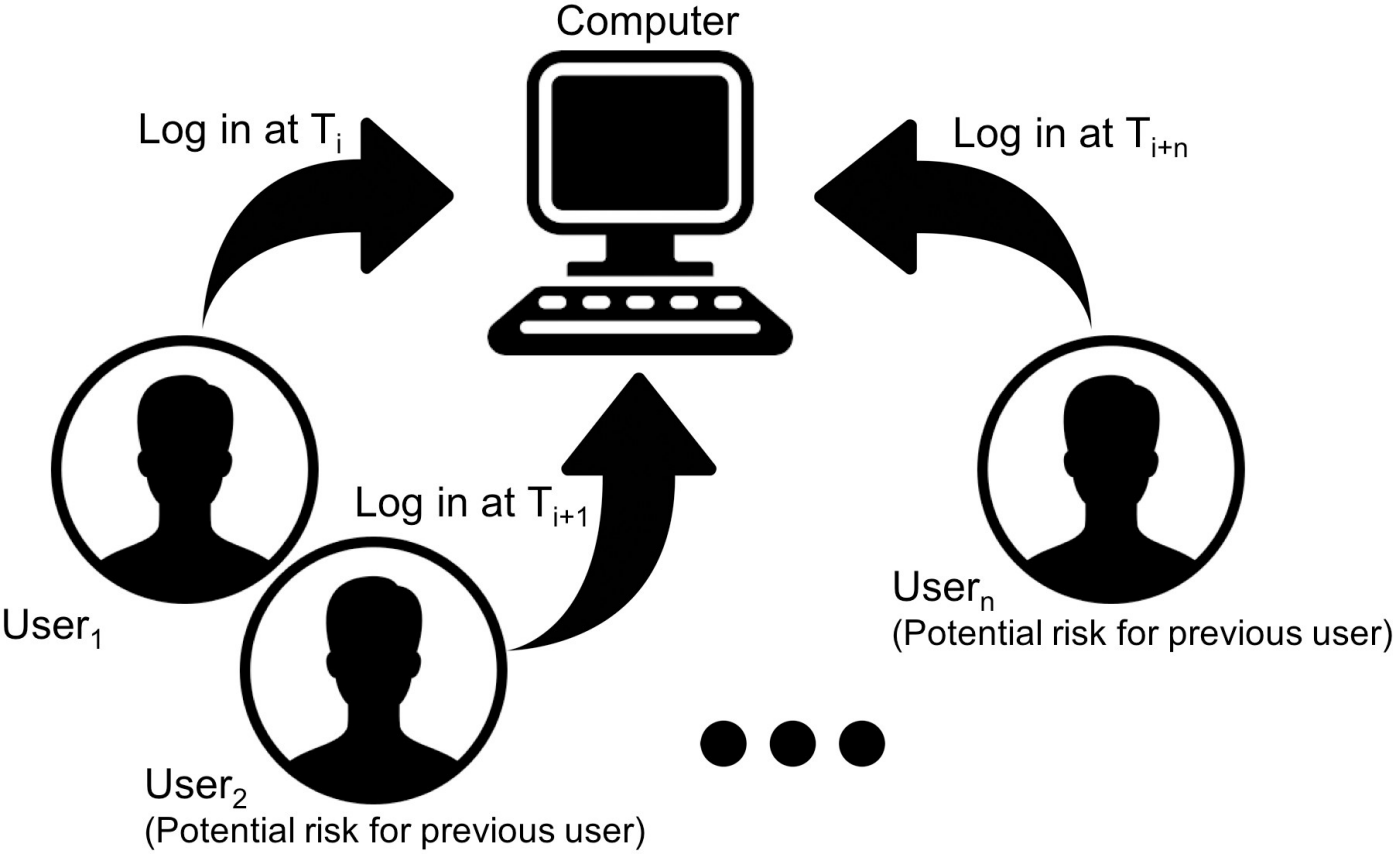
A multi-user computer environment.

Our main assumption, as we stated earlier, is that any users who log in to some computer pose a risk to the data of a user who logged in to the same computer, earlier. This scenario is illustrated in Fig 3.

We call users ${\text{User}}_{2}\ldots {\text{User}}_{n}\in U$ *subsequent users* to ${\text{User}}_{1}\in U$, and we also call the shared computer environment, a *multi-user computer* environment. The definition of User_2_ … User_*n*_ as subsequent to User_1_ stems from the assumption that (*T*_*i*+1_ > *T*_*i*_) ∧…∧ (*T*_*i*+*n*_ > *T*_*i*_). The risk that subsequent users pose may be related to any of the CIA properties and beyond. For example, subsequent users who are not authorised to access the previous users’ data may end up viewing, altering or deleting that data. As a result, determining on average how many such subsequent users there are, provides some measure of the *riskiness* of leaving sensitive data behind when logging in to a multi-user computer. Note we do not consider here risks that arise from previous users, i.e. scenarios where User_1_ may have installed a malicious program that would harm data left on the computer by users User_2_ … User_*n*_. We operate purely based on Locard’s exchange principle [17], which applies only forward in time.

In this section, we aim to answer two specific questions in relation to the UCAAT dataset: *what is the average number of subsequent users on each computer?* and *what is the overall average number of subsequent users in the dataset over all the computers?*, both of which feed into and help us answer the more abstract question, *how do we quantify data visibility?* In order to answer the first question, we start by defining the subsequent users function, *after*, as follows:

$$\begin{array}{c} after:C\times U\to N^{+} \end{array}$$ (1)

which returns a non-negative natural number representing the number of subsequent users who logged in since the first time some user u∈U logged in on a particular computer c∈C. We can define *after* as follows, where UCAAT refers to our datset:

$$\begin{array}{c} after=\{\;\;(c,u,n)\;\;where,\\ \exists t\in N^{+}:\left(t,u,c\right)\in UCAAT\wedge \left(\forall t^{\prime }\in N^{+}:\left(t^{\prime },u,c\right)\in UCAAT\Rightarrow t^{\prime }>t\right)and\\ n=|\{u^{\prime }where,u^{\prime }\neq u\wedge \left(\forall \left(t^{\prime },u^{\prime },c\right)\in UCAAT:t^{\prime }>t\right)\}|\;\;\} \end{array}$$ (2)

This definition states that the *after* relation consists of triples of computer names, user names and the number of subsequent users. The set definition consists of two conditions: the first states that there is a *first* occurrence *in time* (referred to as *t*) of a specific user on a specific computer, and that every other occurrence of that user on that computer is *later* than *t*. This condition acts as a reference to what *t* is. The second condition then defines the number of subsequent users, *n*, by saying that it is the number of other (different) users who logged in on the same computer but at a later time, *t*′. In a data-driven approach, *after* would be defined by the dataset itself. Table 3 represents an example definition of *after* with non-zero subsequent users calculated from the UCAAT dataset for 37 out of the 2808 multi-user computers recorded in the dataset.

**Table 3:** Examples of non-zero numbers of subsequent users in UCAAT.

Computer Name	User Name	Number of Subsequent Users	Computer Name	User Name	Number of Subsequent Users
C10004	U1189	22	C1000	U8525	1
	U713	22		U5724	1
	U1016	22	C10000	U879	8
	U836	22		U43115	7
	U20	22		U516	6
	U2489	22		U6675	5
	U117	22		U4251	4
	U60	22		U6357	2
	U97	22		U8016	2
	U2059	22		U1447	1
	U3316	22	C10003	U2608	6
	U71	22		U265	5
	U156	22		U48	4
	U204	22		U530	3
	U95	22		U8848	2
	U7861	22		U1905	1
	U1234	22	C1013	U92	1
	U4072	22	C10006	U1054	11
	U4975	22		U1143	10
	U8454	21		U1163	9
	U4676	20		U1895	8
	U105	16		U6489	7
	U96	15	C11341	U857	1
C10007	U275	1		U696	6
C10010	U1448	1		U7463	5
C10011	U5734	1		U794	4
C10012	U1448	1		U8050	3
C10019	U4258	1		U858	2
	U8222	1		U8928	1
C1031	U425	1		U9581	1
	U5204	1	C10036	U4258	1
C10039	U4258	1		U5204	1
	U264	1	C10052	U3854	1
C1077	U1847	1	C10074	U8060	1
	U8493	1		U4258	1
C10037	U4258	2		U4258	1
	U5204	2	C10040	U4258	5
	U3692	2		U9392	5
C10041	U4258	2		U9116	4
	U6435	2		U5204	3
	U5204	4		U8988	2
C10043	U4258	1	C10044	U4258	3
	U5204	1		U9692	3
C10046	U4258	2		U9116	4
	U5204	2		U5204	2
	U6392	4		U5434	2
C10047	U4258	3	C10563	U7153	1
	U8619	3	C10051	U8526	2
	U3694	3		U4258	2
	U5204	2		U5204	3
C10020	U4258	1	C10030	U4258	1
	U5382	1		U5204	1
C10005	U1189	30	C10062	U1143	10
	U713	30		U8050	10
	U117	30		U7463	10
	U2489	30		U858	10
	U156	30		U1054	9
	U97	30		U8928	9
	U2059	30		U6489	7
	U60	30		U696	6
	U3316	30		U1895	6
	U1234	30		U794	4
	U1937	30		U1163	5
	U1715	30	C10014	U95	2
	U20	29		U204	2
	U836	29		U8294	2
	U1022	28	C10015	U6675	8
	U7861	28		U4170	8
	U1653	26		U4133	9
	U5580	27		U6466	7
	U1016	26		U5308	4
	U71	26		U5692	2
	U4975	25	C10017	U4258	2
	U204	26		U8721	2
	U95	26		U8321	2
	U6757	23	C10018	U2459	1
	U8338	20	C10028	U4258	1
	U2207	8	C10023	U5204	1

The first two columns represent the domain of the function whereas the third column represents its range. For example, we can see that *after*(C10004, U1189) = 22. This is due to the fact that we find in the dataset the following entries:

(7441936,U1189,C10004)
(7442573,U117,C10004)
(7455652,U97,C10004)
(7458533,U71,C10004)
(7479128,U329,C10004)
(8159950,U99,C10004)
(8183679,U2256,C10004)
(8237229,U156,C10004)
(8239112,U3749,C10004)
(8247201,U60,C10004)
(8264084,U5580,C10004)
(8264505,U3694,C10004)
(8267124,U1715,C10004)
(8584797,U713,C10004)
(8597459,U1,C10004)
(8597975,U105,C10004)
(8605708,U2090,C10004)
(8620034,U10358,C10004)
(9191783,U2489,C10004)
(9198268,U102,C10004)
(9462569,U204,C10004)
(12906381,U96,C10004)
(12915120,U9135,C10004)

where the first entry above represents the first time U1189 logged in on C10004 (at relative time 7441936). The rest of the entries represent the 22 users who logged in on C10004 since then. We do not include in this paper the full calculation of *after* (i.e. for all the 2808 users in the dataset) due to the large space this will take.

Next, we define the average number of subsequent users for some computer through the following function:

$$\begin{array}{c} average:C\to R^{+} \end{array}$$ (3)

which is computed as follows:

$$\begin{array}{c} average\left(c\right):\frac{\sum_{i=1}^{\left|\text{users}(c)\right|}after(c,u_{i})}{\left|\text{users}(c)\right|}\;whereu_{i}\in \text{users}\left(c\right) \end{array}$$ (4)

users:C→℘(U) is a function that accepts a computer name *c* as its parameter and returns the set of all the users who logged on to that computer in the dataset:

$$\begin{array}{c} \text{users}=\{\left(c,\left\{u_{1},\ldots ,u_{n}\right\}\right)\;\;where,\forall u\in \left\{u_{1},\ldots ,u_{n}\right\},\exists t,c:\;\left(t,u,c\right)\in UCAAT\} \end{array}$$ (5)

For example, *users*(C10004) = {U1189, U117, U97, U71, U329, U99, U2256, U156, U3749, U60, U5580, U3694, U1715, U713, U1, U105, U2090, U10358, U2489, U102, U204, U96, U9135}. Again we assume here that |*users*| > 1. The result of computing *average* will be a positive real number representing the average for a particular computer. Table 4 shows an example of the calculation of the *average* function based on the example of Table 3. The average numbers for each computer in the whole dataset are given in S1 File. This so far answers our first question.

**Table 4:** Average number of subsequent users per computer for Table 3.

Computer	Average	Computer	Average	Computer	Average
C10004	20.6	C10007	0.5	C10010	0.5
C10012	0.5	C10019	1.0	C1031	1.0
C10037	2.0	C10041	2.0	C10043	1.0
C10047	2.75	C10005	28.2	C10020	1.0
C1000	0.66	C10000	4.37	C10003	3.5
C10006	7.5	C11341	2.87	C10036	1.0
C10040	3.8	C10044	2.8	C10563	0.5
C10074	0.66	C10014	2.0	C10015	4.22
C10018	0.5	C10028	0.5	C10023	0.5
C10062	7.16	C10011	0.5	C10039	1.0
C10046	2.5	C1077	1	C1013	0.5
C10052	2.0	C10051	1.75	C10017	2.0
C10030	1.0				

In order to answer our second question, however, we need to introduce another function, *aoa*:

$$\begin{array}{c} \text{aoa}:℘\left(C\right)\to R^{+} \end{array}$$ (6)

which takes a set of computers (in this case, the set of multi-user computers in the UCAAT dataset) and returns the average of averages for those computers, as follows:

$$\begin{array}{c} \text{aoa}\left(\text{mucomp}\right)=\frac{\sum_{j=1}^{\left|\text{mucomp}\right|}\left(average\left(c_{j}\right)\right)}{\left|\text{mucomp}\right|}\;wherec_{j}\in \text{mucomp} \end{array}$$ (7)

where mucomp⊆C is the subset of computers, which are multi-user:

$$\begin{array}{c} \text{mucomp}=\{c\;\;where,|\text{users}(c)|>1\} \end{array}$$ (8)

In UCAAT, we have that |*mucomp*| = 2808. The average of averages for UCAAT after calculating the above becomes:

$$\begin{array}{c} \text{aoa}=14.11 \end{array}$$

This means that, on average, there were over 14 users who logged in to a multi-user computer after every user in the UCAAT dataset. From a security risk point of view, one may think of both the *average* and *aoa* values as *metrics* that provide some idea of the possibility that sensitive user data can *flow* to subsequent (unauthorised) users, either from the perspective of a single machine (the *average* metric) or from the perspective of the whole infrastructure of computers in the organisation (the *aoa* metric).

## Security analysis and usability

The next question that we could ask ourselves in the general context of security and information leakage is *how can the metrics above be used to quantify the loss of data security? and how can this quantification be deployed as part of the user’s interface to a computer?* To answer these questions, we first follow the approach defined by [45], who specify the risk of the loss of data privacy in a social media network platform as the product of data visibility and data sensitivity. Data visibility is defined as the number of (potentially unauthorised) users who may have access to the data. We consider our two metrics above as variations of such visibility. Note that we generalise the problem from a data privacy to a data security one, based on the assumption that subsequent users may not only cause the *leak* of sensitive data (i.e. breach privacy and secrecy properties) but also may *alter* the data (i.e. tamper with information integrity) or even *delete* the data altogether (i.e. affect the availability of information).

Data sensitivity, on the other hand, reflects how valuable the data are to their owner (we assume here that the original user who logged in owns their data). We represent this sensitivity as a level that could be assigned as a real number in the range of [0, 1], where 0 represents non-sensitive data and 1 represents ultra sensitive data. One approach for determining this sensitivity level is to assign the value based on the number of security properties required of the data. If we are working within the CIA definition of security, then the number of such properties is 3. More specifically, if data *d* require all three CIA properties, then *d* would be assigned the sensitivity value 1. On the other hand, if the requirement is to only have one property (e.g. confidentiality, integrity or availability), then the sensitivity level would be 1/3 and so on. In a real world-based scenario, such sensitivity should be weighted by the *impact* each property has on the data, which would be derived through an impact case study of that data losing the specific security property. The impact could be defined as a normalised value, in the range [0, 1], where 0 represents no impact and 1 represents maximum impact.

With this in mind, we define the sensitivity of data as a function:

$$\begin{array}{c} \text{sensitivity}:D\times U\to R \end{array}$$ (9)

which, given some data d∈D owned by a user u∈U, it returns a real number representing the sensitivity value for that data. In its general form, this function is calculated as follows:

$$\begin{array}{c} \text{sensitivity}\left(d,u\right)=\left(\left(1/n\right)\times {\text{weight}}_{p1}\left(d,u\right)\right)+\;\ldots \;+\left(\left(1/n\right)\times {\text{weight}}_{pn}\left(d,u\right)\right) \end{array}$$ (10)

where *n* is the number of security properties that data *d* owned by *u* could be sensitive to. The function *weight*_*pi*_(*d*, *u*) for *i* ∈ {1, …, *n*} returns the weight of the security property *pi* in relation to the data and the user. Assuming only CIA properties, this function can be simplified further as follows:

$$\begin{array}{c} \begin{array}{cc} {\text{sensitivity}}_{CIA}\left(d,u\right)= & \left(1/3\times {\text{weight}}_{C}\left(d,u\right)\right)\;+\;\left(1/3\times {\text{weight}}_{I}\left(d,u\right)\right)\;+\;\\ & \left(1/3\times {\text{weight}}_{A}\left(d,u\right)\right) \end{array} \end{array}$$ (11)

Where *weight*_*C*_(*d*, *u*), *weight*_*I*_(*d*, *u*) and *weight*_*A*_(*d*, *u*) are three functions that return the weights of confidentiality, integrity and availability properties, respectively. For simplicity (and in the absence of an impact case study), we set the weight of a property to 0 if the property is not relevant and 1 if it is.

The above definition of data sensitivity is only one example. One could adopt any other different but suitable definition of data sensitivity. One such different example would be based on a lattice structure [63] such as is the case with multilevel security policies [30, 64]. Delving further into data sensitivity definitions is out of the scope of the work presented here. We next use the above definitions of data visibility and sensitivity to define data security loss.

### Loss of data security with user login history

The first definition calculates the potential for the loss of data security directly on the information available from user’s login history. More specifically, we assume that we know per each user, what the number of subsequent users was. This is formalised by the following function:

$$\begin{array}{c} \text{security}\_\text{loss}\_\text{user}:(D\times U\times C)\to R \end{array}$$ (12)

which given some data d∈D owned by a user u∈U who logged in to a computer c∈C, returns a real number representing the potential loss of data security for that user:

$$\begin{array}{c} \text{security}\_\text{loss}\_\text{user}(d,u,c)=\text{sensitivity}(d,u)\times after(c,u) \end{array}$$ (13)

The significance of the value returned by the *security_loss_user*(*d*, *u*, *c*) function is that it represents an overall aggregation of all the possible contexts within which the data may lose their desirable security properties reflected in the potential data security loss value. We consider that each subsequent user represents potentially one such unique context.

Table 5 shows one example of how Eq (13) can be calculated for the case of computer C10000 from Table 3 and for hypothetical data sensitivity values calculated based on the definition of *sensitivity*_*CIA*_. Therefore, data sensitivity values of 0.00 represent insensitive (public) data, 1/3 represent data sensitive to only one CIA property, 2/3 represent data sensitive to two CIA properties and finally, 1.00 represent data sensitive to all CIA properties (ultra sensitive data).

**Table 5:** An example of the calculation of the *security_loss_user* function for Table 3.

User	Number of Subsequent Users	Data Sensitivity Value	Data Security Loss
U879	8	2/3	5.28
U43115	7	1/3	2.31
U516	6	1/3	1.98
U6675	5	2/3	3.3
U4251	4	1/3	1.32
U6357	2	0.00	0.00
U8016	2	2/3	1.32
U1447	1	2/3	0.66

From a usability perspective, such information could lead to the understanding of the potential of data security losses for a computer from its historical data. We can provide this information as a security metadata tag for that computer *C*. This metadata tag could be the average of all the potential data security losses. For example, in Table 5, this average number of potential data security losses would be 2.02 for computer C10000. This information could be used as part of a monitoring process that feeds metadata values into a risk evaluation system, which in turn calculates the risk values for various segments of the network, clustering these into segments of high/medium/low risk.

### Loss of data security for new users and a specific computer

In the second scenario, we assume that a new user is assigned a computer to log in to with no prior history of logging to that machine. In this case, the potential for data security loss is defined through the following function:

$$\begin{array}{c} \text{security}\_\text{loss}\_\text{computer}:(D\times U\times C)\to R \end{array}$$ (14)

which is calculated as follows:

$$\begin{array}{c} \text{security}\_\text{loss}\_\text{computer}(d,u,c)=\text{sensitivity}(d,u)\times average(c) \end{array}$$ (15)

If we consider the same example above for computer C10000, we have that average number of subsequent users for that machine was *averge*(C10000) = 4.37. Therefore, for a new user, *u*_*new*_, whose data sensitivity value is defined as *sensitivity*_*CIA*_(*d*_*new*_, *u*_*new*_) = 2/3 (i.e. as a result of two of the CIA properties being of importance to that data), we have that:

$$\begin{array}{c} \text{security}\_\text{loss}\_\text{computer}(d_{\text{new}},u_{\text{new}},\text{C}10000)=2/3\times 4.37=2.88 \end{array}$$

In a real world access control scenario, this could lead to a new computer log in interface provided for users, where the log in does not only check the user’s identity and password, but also asks the user for the sensitivity value to be provided. The interface would then warn the user of the potential data security loss before proceeding with the log in (assuming the identity and password values are correct). From the new user’s point of view, the user would have a data set (*d*_1_, …, *d*_*n*_), each data with their own sensitivity level, (*sensitivity*(*d*_1_, *u*_*new*_), …, *sensitivity*(*d*_*n*_, *u*_*new*_)). The user then may have a security policy constraint that imposes a maximum loss value ℓ∈R on computer *c* that the user is logging into:

$$\begin{array}{c} \begin{array}{cc} & (\left(\text{security}\_\text{loss}\_\text{computer}\left(d_{1},u_{\text{new}},c\right)\leq \ell \right)\;\wedge \;\ldots \;\wedge \;\\ & \left(\text{security}\_\text{loss}\_\text{computer}\left(d_{n},u_{\text{new}},c\right)\leq \ell )\right) \end{array} \end{array}$$ (16)

This level *ℓ* represents the user’s level of tolerance with regards to the potential of losing their data security.

Returning to our example, assume the user has data $\left(d_{\text{new}},d_{\text{new}}^{\prime },d_{\text{new}}^{\prime \prime }\right)$ with sensitivity values for example:

*sensitivity*_*CIA*_(*d*_*new*_, *u*_*new*_) = 2/3

${\text{sensitivity}}_{CIA}\left(d_{\text{new}}^{\prime },u_{\text{new}}\right)=1.00$

${\text{sensitivity}}_{CIA}\left(d_{\text{new}}^{\prime \prime },u_{\text{new}}\right)=1/3$

and the user has set their tolerance level *ℓ* = 4, then the constraint for this example would evaluate to:

((*security_loss_computer*(*d*, *u*_*new*_, C10000) ≤ 4.00) ∧
(*security_loss_computer*(*d*′, *u*_*new*_, C10000) ≤ 4.00) ∧
(*security_loss_computer*(*d*′′, *u*_*new*_, C10000) ≤ 4.00)) =
((2.88 ≤ 4.00) ∧ (4.37 ≤ 4.00) ∧ (1.44 ≤ 4.00)) = False

Therefore, the user may decide not to log in to computer C10000, and perhaps choose to log in to another, less risky, computer.

### Loss of data security for new users and no specific computer

The third and final definition of the potential for the loss of data security assumes that no specific computer is assigned to a new user and that the user may log in to any computer in the organisation. This potential loss is defined by the following function:

$$\begin{array}{c} \text{security}\_\text{loss}:(D\times U\times ℘(C))\to R \end{array}$$ (17)

which takes the user and the sensitivity level of the user’s data, as well as the overall set of the multi-user computers that that user may log in to, and returns a number representing the potential of the loss of data security. This is calculated as follows:

$$\begin{array}{c} \text{security}\_\text{loss}(d,u,\text{mucomp})=\text{sensitivity}(d,u)\times \text{aoa}(\text{mucomp}) \end{array}$$ (18)

In the case of the UCAAT set of computers, this equation can be simplified to:

$$\begin{array}{c} \text{security}\_{\text{loss}}_{UCAAT}\left(d,u\right)=\text{sensitivity}\left(d,u\right)\times 14.11 \end{array}$$ (19)

A new user may use Eq (19) to express their constraint on the potential data security loss in UCAAT computers, as follows:

$$\begin{array}{c} \begin{array}{cc} & (\left(\text{security}\_{\text{loss}}_{UCAAT}\left(d_{1},u_{\text{new}}\right)\leq \ell \right)\;\wedge \;\ldots \;\wedge \;\\ & \left(\text{security}\_{\text{loss}}_{UCAAT}\left(d_{n},u_{\text{new}}\right)\leq \ell )\right) \end{array} \end{array}$$ (20)

For example, using the same data set of the previous section, the constraint becomes:

((*security_loss*_*UCAAT*_(*d*_1_, *u*_*new*_) ≤ 4.00) ∧
(*security_loss*_*UCAAT*_(*d*_2_, *u*_*new*_) ≤ 4.00) ∧
(*security_loss*_*UCAAT*_(*d*_3_, *u*_*new*_) ≤ 4.00)) =
((9.31 ≤ 4.00) ∧ (14.11 ≤ 4.00) ∧ (4.66 ≤ 4.00)) = False

This result will warn a new user that the UCAAT set of computers is, in general, too risky for the data set that that user owns and that the user may end up storing (either intentionally or unintentionally) on those computers.

## Conclusion

We presented in this paper a data-drive research framework for the analysis of an example dataset that associates users authentication to individual computers. The analysis aimed at defining the visibility of users datasets that may be accessed by subsequent and possibly unauthorised users logging in to the same computers. We discussed how this information, when coupled with data sensitivity information, can define a useful new data security-oriented log in interface to assist users in making the decision as to whether or not a specific computer or the whole IT infrastructure is risky before they attempt to log in.

In the future, we plan as a first direction of research, to implement the new computer interface and conduct an empirical study to understand its impact on reducing data security losses in organisations. Another direction for future research would be to pose other, possibly more complex, research questions related to the UCAAT dataset in order to understand if this dataset contains other security-related information. The answers to such questions would lead to different log in interfaces. The validity of the current subsequent users analysis itself can be more rigorously tested and evaluated, for example, through testing the analysis on a variety of other datasets and comparing the results to the existing analysis. Additionally, the current analysis itself can be applied to different other scenarios, where users are associated with computers or indeed, any other computing device. We plan to investigate some other scenarios, e.g. in a university computer laboratory setting, to understand whether a risk-driven to user-computer associations has any impact on users’ behaviour.

## Supporting information

S1 File A Average number *n* of subsequent users in UCAAT [18, 19]. (PDF)
