# Supplementary material for: Analysing potential data security losses in organisations based on subsequent users logins
Source: PLoS One. 2023 Aug 24;18(8):e0286856. doi: 10.1371/journal.pone.0286856 (PMC10449169; doi:10.1371/journal.pone.0286856)
Supplement: S1 File — (PDF) [file pone.0286856.s001.pdf]

## A Average number $n$ of subsequent users in UCAAT

The following Table 6 shows the average number $n$ of subsequent users that log in after each user on the UCAAT computers [18, 19]. This represents the extended version of the subsequent users analysis summarised in Section Subsequent Users Analysis.

| Computer | n | Computer | n | Computer | n | Computer | n |
|---|---|---|---|---|---|---|---|
| C1 | 63.11 | C10004 | 20.6 | C10007 | 0.5 | C11156 | 1 |
| C1000 | 0.66 | C10000 | 4.37 | C100003 | 3 | C11163 | 1 |
| C1013 | 0.5 | C10006 | 7.5 | C10005 | 28.2 | C11165 | 1 |
| C10014 | 2 | C10015 | 4.22 | C10016 | 5.2 | C11166 | 0.5 |
| C10017 | 2 | C10018 | 0.5 | C10028 | 0.5 | C11167 | 2 |
| C10020 | 1 | C10021 | 2.75 | C10023 | 0.5 | C11168 | 0.5 |
| C10024 | 0.66 | C10025 | 2.75 | C10026 | 1.5 | C11175 | 0.5 |
| C10027 | 1.5 | C10029 | 2.8 | C10030 | 1 | C11177 | 1 |
| C10031 | 0.66 | C10035 | 2.25 | C10036 | 1 | C11178 | 1 |
| C10039 | 1 | C10040 | 3.8 | C10044 | 2.8 | C11180 | 2.5 |
| C10563 | 0.5 | C10051 | 1.75 | C1005 | 1.66 | C11181 | 1 |
| C10054 | 0.66 | C10058 | 4.11 | C10062 | 7.16 | C11183 | 3.5 |
| C10074 | 0.66 | C10079 | 0.66 | C10081 | 2.25 | C11185 | 0.5 |
| C10088 | 0.66 | C10089 | 5.8 | C10084 | 3 | C11186 | 1.5 |
| C10095 | 0.66 | C10196 | 2.4 | C10198 | 2 | C11187 | 0.5 |
| C10202 | 2 | C10208 | 0.66 | C10205 | 0.66 | C11189 | 1 |
| C10207 | 2 | C10217 | 0.66 | C10216 | 3 | C11191 | 0.5 |
| C10220 | 2.5 | C10221 | 0.66 | C10226 | 1 | C11193 | 0.5 |
| C10238 | 4.33 | C10230 | 3 | C10241 | 0.66 | C11195 | 0.5 |
| C10242 | 1.33 | C10246 | 0.66 | C10249 | 2 | C11198 | 0.5 |
| C10264 | 0.66 | C10266 | 0.66 | C10271 | 0.66 | C112 | 23.02 |
| C10277 | 0.66 | C10284 | 0.66 | C10285 | 0.66 | C11202 | 2.1 |
| C10289 | 0.66 | C10293 | 3.2 | C10297 | 2.2 | C11209 | 0.5 |
| C10304 | 2 | C10306 | 0.66 | C10308 | 2 | C1121 | 0.5 |
| C1031 | 1 | C10244 | 1.33 | C10248 | 0.66 | C11211 | 0.5 |
| C10257 | 1 | C10262 | 2.5 | C10027 | 0.66 | C11214 | 3.5 |
| C10273 | 0.66 | C10275 | 6 | C10286 | 1.72 | C11219 | 0.5 |
| C10290 | 0.66 | C10294 | 2.25 | C103 | 2.75 | C11122 | 0.5 |
| C10313 | 9 | C10315 | 2 | C10318 | 3 | C1122 | 1 |
| C10322 | 2.5 | C10323 | 2 | C10332 | 0.66 | C11226 | 0.5 |
| C10335 | 0.66 | C10341 | 0.66 | C10342 | 0.66 | C11228 | 0.5 |
| C10347 | 1.5 | C10351 | 3 | C10357 | 0.66 | C11233 | 0.5 |
| C4704 | 8.5 | C4706 | 19 | C4708 | 49.5 | C4709 | 5 |
| C471 | 6.5 | C4710 | 40.6 | C4712 | 19.6 | C4713 | 26 |
| C4714 | 5.5 | C4715 | 2.5 | C4716 | 8.5 | C4718 | 1 |

**Table 6.** Average number $n$ of subsequent users per multi-user computer

| Computer | n | Computer | n | Computer | n | Computer | n |
|---|---|---|---|---|---|---|---|
| C10361 | 3.6 | C10371 | 0.66 | C10372 | 1.75 | C11274 | 0.5 |
| C10376 | 2 | C10379 | 0.66 | C10384 | 1.66 | C11276 | 0.5 |
| C10389 | 0.66 | C10393 | 0.66 | C10394 | 0.66 | C11277 | 1 |
| C10402 | 2 | C10404 | 4 | C10422 | 0.66 | C11283 | 0.5 |
| C10424 | 0.66 | C10431 | 0.66 | C10442 | 0.66 | C11285 | 1.5 |
| C10445 | 0.66 | C10321 | 4.77 | C10324 | 3 | C11288 | 1 |
| C10329 | 1.66 | C10336 | 4.77 | C10345 | 0.66 | C11289 | 1 |
| C10349 | 1.66 | C10352 | 1.5 | C10359 | 1 | C1129 | 1.6 |
| C10362 | 0.66 | C10365 | 4.77 | C10368 | 3 | C11293 | 1.5 |
| C10374 | 0.66 | C10378 | 2 | C10381 | 0.66 | C11296 | 0.5 |
| C10388 | 0.66 | C10391 | 0.66 | C10394 | 1.66 | C11297 | 0.5 |
| C104 | 4.33 | C1041 | 0.66 | C10412 | 0.66 | C113 | 7.7 |
| C10423 | 0.66 | C10428 | 0.66 | C10440 | 0.66 | C11304 | 0.5 |
| C10382 | 3.66 | C10410 | 10.76 | C10429 | 3 | C11308 | 0.5 |
| C10453 | 2.66 | C10458 | 4.28 | C10468 | 2.25 | C11309 | 0.5 |
| C10475 | 0.66 | C10477 | 2.75 | C10479 | 0.66 | C1131 | 0.5 |
| C10484 | 0.66 | C10492 | 0.66 | C10499 | 0.66 | C11313 | 1.5 |
| C105 | 6 | C10500 | 0.5 | C10503 | 0.5 | C11314 | 1 |
| C10504 | 2 | C10506 | 9.6 | C10507 | 7.07 | C11316 | 0.5 |
| C10509 | 3 | C10551 | 0.5 | C10511 | 0.5 | C11318 | 0.5 |
| C10513 | 0.5 | C10514 | 0.5 | C10515 | 2.25 | C11319 | 0.5 |
| C10518 | 2 | C10519 | 0.5 | C1052 | 5.6 | C11321 | 1.5 |
| C10522 | 0.5 | C10527 | 0.5 | C1053 | 10.9 | C11323 | 1.7 |
| C10539 | 4 | C10541 | 4 | C10542 | 0.5 | C11324 | 2.5 |
| C10544 | 3 | C10545 | 2.6 | C10547 | 0.5 | C11334 | 0.5 |
| C10548 | 1.5 | C1055 | 0.5 | C10550 | 0.5 | C11330 | 0.5 |
| C10552 | 4 | C10554 | 2.4 | C10555 | 0.5 | C11332 | 0.5 |
| C10563 | 0.5 | C10507 | 33.8 | C10570 | 1 | C11334 | 1 |
| C10573 | 2 | C10577 | 0.5 | C10558 | 1 | C11336 | 1.5 |
| C10580 | 1 | C10583 | 1 | C10584 | 1 | C11341 | 2.87 |
| C10580 | 0.5 | C10591 | 1 | C10592 | 1.6 | C11344 | 1.5 |
| C10593 | 2 | C10594 | 2.5 | C10597 | 1.6 | C11345 | 1 |
| C4683 | 3 | C4685 | 38 | C4687 | 1 | C4688 | 1 |
| C4689 | 24.5 | C469 | 8 | C4690 | 2.5 | C4691 | 20.5 |
| C4692 | 43 | C4694 | 9.5 | C4695 | 1 | C4696 | 16.4 |
| C4697 | 7 | C4700 | 7.5 | C4701 | 13.1 | C4702 | 5 |
| C4628 | 1.5 | C4633 | 1.5 | C4651 | 1.7 | C4671 | 5.5 |

**Table 6.** Average number $n$ of subsequent users per multi-user computer (continued)

| Computer | n | Computer | n | Computer | n | Computer | n |
|---|---|---|---|---|---|---|---|
| C10842 | 0.5 | C10843 | 1 | C10845 | 2 | C11386 | 1 |
| C10848 | 1 | C10852 | 1 | C10855 | 1.5 | C11387 | 7.5 |
| C10857 | 1 | C10860 | 2.2 | C10862 | 2.1 | C11388 | 0.5 |
| C10870 | 1 | C10871 | 3 | C10872 | 1 | C11389 | 0.5 |
| C10873 | 0.5 | C10875 | 0.5 | C1088 | 3.5 | C1139 | 97.5 |
| C10880 | 2.5 | C10884 | 1 | C10888 | 1.5 | C11391 | 1 |
| C10889 | 1.5 | C1089 | 0.5 | C10895 | 0.5 | C11392 | 0.5 |
| C109 | 1 | C1090 | 3.5 | C10903 | 1 | C11394 | 1 |
| C10907 | 1.5 | C10910 | 1.5 | C10911 | 1.5 | C11395 | 3 |
| C10914 | 1.5 | C10917 | 1 | C1092 | 1 | C11397 | 0.5 |
| C10921 | 0.5 | C109221 | 0.5 | C10923 | 0.5 | C11398 | 0.5 |
| C10926 | 4 | C10927 | 1 | C10931 | 1 | C11398 | 0.5 |
| C10932 | 1 | C10933 | 0.5 | C10934 | 1 | C1134 | 0.5 |
| C10935 | 3.6 | C10936 | 0.5 | C10937 | 0.5 | C114 | 237.5 |
| C10938 | 1 | C10939 | 3 | C10940 | 0.5 | C11404 | 3 |
| C10943 | 0.5 | C10946 | 1 | C10947 | 1 | C11408 | 0.5 |
| C10949 | 0.5 | C1095 | 55 | C10951 | 0.5 | C11409 | 0.5 |
| C10956 | 2 | C10959 | 0.5 | C10960 | 1 | C11411 | 0.5 |
| C10961 | 0.5 | C10968 | 1 | C1097 | 15.5 | C11413 | 0.5 |
| C10971 | 1.5 | C10972 | 1 | C10974 | 2.1 | C11416 | 2.5 |
| C1098 | 13.7 | C10978 | 0.5 | C10980 | 0.5 | C11417 | 2.5 |
| C10981 | 0.5 | C10984 | 2 | C10986 | 0.5 | C11418 | 0.5 |
| C10987 | 0.5 | C10989 | 2 | C10991 | 0.5 | C1142 | 0.5 |
| C10992 | 0.5 | C10993 | 0.5 | C10996 | 0.5 | C11420 | 0.5 |
| C10998 | 1 | C11 | 2 | C110 | 2.7 | C11421 | 0.5 |
| C11001 | 2.5 | C11002 | 3 | C11003 | 0.5 | C11424 | 1.5 |
| C11008 | 0.5 | C11010 | 4.8 | C11013 | 2.2 | C11426 | 0.5 |
| C11014 | 5.5 | C11023 | 0.5 | C11024 | 0.5 | C11427 | 1 |
| C11026 | 0.5 | C11027 | 4.5 | C1103 | 0.5 | C11428 | 3 |
| C4532 | 1.5 | C4544 | 6 | C4552 | 0.5 | C4553 | 0.5 |
| C4555 | 564 | C456 | 56.5 | C4560 | 6 | C4561 | 1.5 |
| C4573 | 1.5 | C4582 | 1 | C4585 | 1 | C459 | 20 |
| C4602 | 52 | C4605 | 169 | C4612 | 8 | C4619 | 1 |
| C4672 | 3 | C4677 | 44 | C4680 | 1.4 | C4681 | 13.5 |
| C2954 | 0.5 | C2958 | 1 | C2967 | 0.5 | C2969 | 29.5 |
| C2974 | 1.5 | C298 | 0.5 | C299 | 0.5 | C2993 | 15.5 |
| C2934 | 1 | C2937 | 284.5 | C2945 | 8.5 | C295 | 9 |

**Table 6.** Average number $n$ of subsequent users per multi-user computer (continued)

| Computer | n | Computer | n | Computer | n | Computer | n |
|---|---|---|---|---|---|---|---|
| C11484 | 1.5 | C11485 | 0.5 | C11486 | 1 | C11487 | 1 |
| C11489 | 0.5 | C1149 | 0.5 | C11490 | 1 | C11494 | 1.5 |
| C11496 | 0.5 | C11498 | 1 | C115 | C300 | C11500 | 4.5 |
| C11509 | 6 | C11511 | 1 | C11512 | 0.5 | C11514 | 1.5 |
| C11517 | 0.5 | C11519 | 0.5 | C11512 | 0.5 | C11521 | 0.5 |
| C11522 | 2 | C11524 | 2.5 | C11529 | 0.5 | C11532 | 0.5 |
| C11535 | 0.5 | C11540 | 0.5 | C11542 | 1 | C1155 | 0.5 |
| C11551 | 0.5 | C11552 | 2.5 | C11555 | 0.5 | C11558 | 0.5 |
| C11560 | 1.5 | C11562 | 0.5 | C11562 | 0.5 | C11563 | 1.5 |
| C11564 | 0.5 | C11565 | 0.5 | C11566 | 1.5 | C11571 | 0.5 |
| C11577 | 1.5 | C11578 | 0.5 | C11580 | 2 | C11581 | 0.5 |
| C11582 | 1.5 | C11594 | 7 | C11597 | 3.5 | C11599 | 5.5 |
| C116 | 6.5 | C11601 | 4.5 | C11580 | 2 | C11602 | 0.5 |
| C11603 | 5 | C11605 | 3.6 | C11606 | 0.5 | C11607 | 2.4 |
| C11608 | 5 | C11578 | 0.5 | C11610 | 4 | C11611 | 3.5 |
| C11612 | 0.5 | C11614 | 2.4 | C11615 | 3 | C11617 | 4 |
| C11619 | 1 | C11624 | 2.2 | C11625 | 5 | C11627 | 0.5 |
| C11636 | 1.5 | C11642 | 4.5 | C11644 | 3 | C11649 | 2 |
| C11620 | 2 | C11651 | 1.5 | C11653 | 1 | C11658 | 1.5 |
| C11663 | 0.5 | C11667 | 0.5 | C11668 | 0.5 | C11673 | 0.5 |
| C11674 | 0.5 | C11676 | 0.5 | C11679 | 1 | C11681 | 0.5 |
| C11682 | 1 | C11684 | 1 | C11686 | 2.5 | C11691 | 0.5 |
| C11696 | 1 | C11697 | 3 | C117 | 3.5 | C11704 | 0.5 |
| C11706 | 1 | C11708 | 1.5 | C11709 | 2.5 | C11710 | 3.5 |
| C11721 | 1.4 | C11733 | 2.5 | C11736 | 1.5 | C11738 | 0.5 |
| C11739 | 0.5 | C11747 | 0.5 | C11750 | 1.5 | C11751 | 0.5 |
| C11754 | 1 | C11756 | 0.5 | C11709 | 2.5 | C11771 | 1.5 |
| C11773 | 0.5 | C117778 | 0.5 | C11778 | 2.5 | C11782 | 2 |
| C11788 | 1 | C11792 | 1 | C11793 | 1 | C11794 | 1 |
| C11801 | 1 | C11802 | 1 | C11803 | 3 | C11808 | 1 |
| C11815 | 0.5 | C1182 | 57 | C11823 | 3.5 | C11824 | 2 |
| C11833 | 4.5 | C11834 | 1.5 | C11835 | 1 | C11837 | 0.5 |
| C3383 | 21 | C3409 | 6.5 | C3412 | 2.5 | C3417 | 25 |
| C342 | 1.5 | C3423 | 0.5 | C3424 | 0.5 | C3431 | 1 |
| C2906 | 15.5 | C292 | 16.1 | C2891 | 6 | C293 | 50 |
| C3293 | 11.5 | C3294 | 8 | C330 | 4.5 | C3337 | 1 |
| C3350 | 4 | C336 | 1 | C3368 | 287 | C3369 | 0.5 |

**Table 6.** Average number $n$ of subsequent users per multi-user computer (continued)

| Computer | n | Computer | n | Computer | n | Computer | n |
|---|---|---|---|---|---|---|---|
| C12084 | 1.5 | C12086 | 0.5 | C12088 | 2 | C1209 | 0.5 |
| C12090 | 1.7 | C12091 | 1 | C12092 | 1 | C12095 | 2.5 |
| C12096 | 1 | C12097 | 0.5 | C12098 | 1 | C1210 | 113.5 |
| C12100 | 0.5 | C12102 | 0.5 | C12104 | 0.5 | C12105 | 2 |
| C12107 | 0.5 | C12108 | 4.5 | C12109 | 0.5 | C1211 | 1 |
| C12111 | 0.5 | C12113 | 0.5 | C12114 | 1 | C12114 | 1 |
| C12121 | 0.5 | C12124 | 0.5 | C12125 | 0.5 | C12126 | 0.5 |
| C12127 | 01.5 | C1213 | 2 | C12130 | 1 | C12131 | 1.5 |
| C12132 | 0.5 | C12133 | 0.5 | C12134 | 0.5 | C12136 | 1 |
| C12137 | 0.5 | C12140 | 0.5 | C12144 | 0.5 | C12150 | 0.5 |
| C12152 | 1 | C12156 | 2 | C12157 | 1 | C12159 | 0.5 |
| C12161 | 0.5 | C12162 | 0.5 | C12164 | 1.5 | C12166 | 2.5 |
| C12169 | 2.5 | C12174 | 1 | C12175 | 1 | C12176 | 0.5 |
| C12180 | 1 | C12182 | 0.5 | C12183 | 1 | C12184 | 0.5 |
| C12185 | 0.5 | C12186 | 0.5 | C12196 | 3.5 | C12200 | 1 |
| C12206 | 0.5 | C12208 | 1 | C12211 | 0.5 | C12212 | 0.5 |
| C12213 | 0.5 | C12215 | 0.5 | C12215 | 0.5 | C12217 | 0.5 |
| C12219 | 0.5 | C12220 | 0.5 | C12221 | 1 | C12223 | 0.5 |
| C12224 | 0.5 | C12225 | 1 | C12226 | 19.2 | C12227 | 0.5 |
| C12232 | 5.5 | C12220 | 0.5 | C1224 | 0.5 | C12241 | 0.5 |
| C12244 | 1 | C12247 | 1 | C12250 | 0.5 | C12254 | 4 |
| C12259 | 0.5 | C12261 | 1 | C12262 | 3.25 | C12263 | 1.5 |
| C12264 | 0.5 | C12268 | 8.5 | C12270 | 0.5 | C12272 | 0.5 |
| C12281 | 5 | C12282 | 4.5 | C12286 | 0.5 | C12292 | 0.5 |
| C12293 | 0.5 | C12294 | 0.5 | C12296 | 0.5 | C1230 | 0.5 |
| C12305 | 18 | C12306 | 0.5 | C12307 | 0.5 | C12311 | 0.5 |
| C12314 | 0.5 | C12232 | 1 | C12320 | 0.5 | C12321 | 1 |
| C12323 | 1.5 | C1233 | 8.5 | C12332 | 0.5 | C12343 | 0.5 |
| C12344 | 1.5 | C12347 | 1.5 | C12354 | 1.5 | C1236 | 0.5 |
| C12361 | 0.5 | C12364 | 0.5 | C12366 | 1 | C1237 | 0.5 |
| C12378 | 0.5 | C1238 | 81.6 | C124 | 1.5 | C12401 | 0.5 |
| C12402 | 1.5 | C12403 | 1 | C12407 | 1.5 | C12414 | 0.5 |
| C3130 | 576 | C3131 | 10 | C3139 | 5 | C3141 | 5 |
| C3146 | 6.5 | C3158 | 6 | C3139 | 5 | C3166 | 7 |
| C317 | 5.5 | C3192 | 6.8 | C3193 | 345 | C3202 | 1 |
| C324 | 1.5 | C3260 | 2.6 | C3262 | 5 | C3268 | 1.5 |
| C3127 | 21 | C3129 | 15.4 | C313 | 2 | C3130 | 882 |

**Table 6.** Average number $n$ of subsequent users per multi-user computer (continued)

| Computer | n | Computer | n | Computer | n | Computer | n |
|---|---|---|---|---|---|---|---|
| C12702 | 0.5 | C12704 | 3 | C12711 | 0.5 | C12714 | 0.5 |
| C12715 | 0.5 | C12718 | 0.5 | C12719 | 0.5 | C12720 | 0.5 |
| C12724 | 1.5 | C12726 | 0.5 | C12727 | 2.5 | C12729 | 0.5 |
| C12735 | 0.5 | C12740 | 0.5 | C12743 | 0.5 | C12746 | 0.5 |
| C12748 | 0.5 | C12749 | 1 | C1275 | 1 | C12750 | 1 |
| C12755 | 0.5 | C12756 | 2.4 | C12763 | 1 | C12765 | 1.5 |
| C12766 | 0.5 | C12767 | 1 | C1277 | 2.8 | C12775 | 1.9 |
| C12778 | 2.5 | C12779 | 0.5 | C12783 | 0.5 | C12784 | 0.5 |
| C12790 | 1 | C12793 | 1 | C12797 | 0.5 | C128 | 24.1 |
| C12801 | 0.5 | C12802 | 0.5 | C12803 | 0.5 | C12809 | 0.5 |
| C12810 | 1 | C12816 | 1.5 | C12818 | 0.5 | C12819 | 1 |
| C1282 | 0.5 | C12821 | 1.5 | C12823 | 0.5 | C12825 | 1 |
| C12827 | 2.5 | C12830 | 1.5 | C12831 | 0.5 | C12832 | 0.5 |
| C12833 | 0.5 | C12835 | 1 | C12837 | 1 | C12839 | 1 |
| C1284 | 1 | C12840 | 0.5 | C12841 | 1 | C12846 | 0.5 |
| C12847 | 1.5 | C12848 | 0.5 | C12849 | 1 | C1285 | 0.5 |
| C12850 | 0.5 | C12853 | 0.5 | C12855 | 1 | C12856 | 3 |
| C12857 | 1 | C12862 | 1 | C12863 | 1 | C12865 | 1.5 |
| C12868 | 0.5 | C12870 | 0.5 | C12871 | 1 | C12874 | 1.5 |
| C12875 | 2 | C12877 | 0.5 | C12882 | 0.5 | C12883 | 0.5 |
| C12884 | 0.5 | C12889 | 0.5 | C12890 | 1 | C12892 | 13 |
| C12893 | 1 | C12895 | 1.5 | C12896 | 1 | C12898 | 0.5 |
| C12899 | 0.5 | C129 | 14 | C12903 | 0.5 | C12908 | 1 |
| C12909 | 0.5 | C12910 | 0.5 | C12911 | 1 | C12913 | 1 |
| C12914 | 1.5 | C12915 | 1 | C12916 | 0.5 | C12919 | 0.5 |
| C12920 | 1.5 | C12922 | 0.5 | C12924 | 0.5 | C12926 | 1 |
| C12927 | 0.5 | C12929 | 1.5 | C12931 | 0.5 | C12933 | 1 |
| C12938 | 1 | C1294 | 1.5 | C12942 | 1 | C12951 | 2.5 |
| C12955 | 1 | C12959 | 5 | C1296 | 0.5 | C12961 | 1.5 |
| C12964 | 0.5 | C12968 | 1.5 | C12979 | 1 | C12983 | 1 |
| C12984 | 1.5 | C12988 | 3 | C12989 | 1 | C12990 | 1 |
| C2999 | 1.5 | C3 | 1.5 | C300 | 86 | C3002 | 1.5 |
| C301 | 800 | C3017 | 7 | C3033 | 39 | C304 | 53 |
| C3040 | 2 | C3042 | 1.5 | C3042 | 1.5 | C3043 | 42.4 |
| C3049 | 144 | C305 | 6 | C309 | 9.5 | C310 | 882 |
| C6743 | 0.5 | C6754 | 84.5 | C6759 | 1 | C677 | 1 |
| C6780 | 5.5 | C6784 | 0.5 | C6791 | 0.5 | C6800 | 0.5 |

**Table 6.** Average number $n$ of subsequent users per multi-user computer (continued)

| Computer | n | Computer | n | Computer | n | Computer | n |
|---|---|---|---|---|---|---|---|
| C9999 | 7.5 | C9998 | 1 | C9996 | 5.5 | C9995 | 5.5 |
| C9993 | 1 | C9992 | 3.4 | C9990 | 3.5 | C9989 | 3.4 |
| C9988 | 0.5 | C9985 | 8 | C9986 | 0.5 | C9983 | 6.5 |
| C9982 | 9 | C9977 | 5.5 | C9973 | 3.5 | C9971 | 3.5 |
| C9969 | 6.1 | C9966 | 5 | C9962 | 1 | C9961 | 9 |
| C9960 | 0.5 | C9958 | 7.5 | C9957 | 4 | C9956 | 3 |
| C9954 | 6.5 | C9953 | 1 | C9952 | 0.5 | C9950 | 0.5 |
| C9947 | 0.5 | C9942 | 4 | C9935 | 0.5 | C9934 | 0.5 |
| C9933 | 0.5 | C9932 | 1 | C9930 | 4.5 | C9929 | 0.5 |
| C9925 | 0.5 | C9922 | 2.5 | C9920 | 0.5 | C9919 | 0.5 |
| C9914 | 1.5 | C99313 | 3.6 | C9910 | 1.6 | C9907 | 1 |
| C9902 | 2.5 | C9901 | 1.5 | C9900 | 1.5 | C9899 | 1.5 |
| C9898 | 0.5 | C9894 | 1 | C9888 | 3.5 | C9882 | 0.5 |
| C9877 | 1.5 | C9870 | 1 | C9868 | 4 | C9859 | 1 |
| C9857 | 1.5 | C9855 | 1 | C9851 | 1.5 | C9846 | 0.5 |
| C9837 | 4 | C9836 | 1.5 | C9834 | 1.5 | C9834 | 3 |
| C9824 | 1.5 | C9811 | 3.5 | C9804 | 1 | C9798 | 1.5 |
| C9797 | 0.5 | C9796 | 6.1 | C9792 | 2.5 | C9789 | 1.5 |
| C9782 | 1.5 | C9780 | 1 | C978 | 4 | C977 | 28.5 |
| C975 | 1 | C9729 | 0.5 | C9719 | 5.5 | C9715 | 1.5 |
| C9714 | 5 | C9711 | 0.5 | C9708 | 0.5 | C9702 | 1.5 |
| C97 | 8 | C9694 | 3.1 | C9691 | 1.5 | C9688 | 0.5 |
| C96 | 3 | C9595 | 2.8 | C9582 | 17.5 | C9572 | 4.5 |
| C957 | 9 | C9567 | 3.2 | C9564 | 3 | C9561 | 2.1 |
| C9553 | 6.5 | C9530 | 4.5 | C9521 | 4.2 | C9518 | 1.6 |
| C95 | 12.5 | C9490 | 10 | C9488 | 6.5 | C9477 | 4 |
| C9461 | 5 | C9530 | 4.5 | C9521 | 4.2 | C9518 | 85.5 |
| C9422 | 22.5 | C9409 | 10 | C94 | 10.5 | C9366 | 8 |
| C93 | 4 | C9262 | 4.5 | C9261 | 42.5 | C9250 | 3.6 |
| C92 | 7.5 | C9108 | 31 | C91 | 4.5 | C9065 | 22 |
| C90 | 5.5 | C8994 | 25 | C8977 | 11 | C8976 | 8 |
| C6554 | 0.5 | C6563 | 0.5 | C6565 | 4 | C6570 | 0.5 |
| C6584 | 3.6 | C659 | 4 | C6597 | 2 | C6599 | 4 |
| C6614 | 0.5 | C6625 | 0.5 | C6638 | 1 | C6656 | 0.5 |
| C6674 | 0.5 | C6682 | 0.5 | C6688 | 1 | C6699 | 1.5 |
| C670 | 344 | C6705 | 1.8 | C6701 | 0.5 | C6724 | 1 |
| C10816 | 4 | C10818 | 0.5 | C10819 | 1.5 | C11263 | 1 |

**Table 6.** Average number $n$ of subsequent users per multi-user computer (continued)

| Computer | n | Computer | n | Computer | n | Computer | n |
|---|---|---|---|---|---|---|---|
| C13298 | 1.5 | C133 | 1 | C13304 | 1.5 | C13311 | 1 |
| C13314 | 1.5 | C13319 | 1 | C13320 | 3 | C13324 | 0.5 |
| C13327 | 0.5 | C13330 | 0.5 | C13332 | 0.5 | C13335 | 1 |
| C13338 | 2 | C13341 | 0.5 | C13347 | 1 | C13353 | 0.5 |
| C13356 | 2 | C13357 | 1.5 | C13361 | 0.5 | C13362 | 0.5 |
| C13366 | 0.5 | C1337 | 3.5 | C13374 | 0.5 | C13376 | 0.5 |
| C13381 | 0.5 | C13386 | 1 | C13389 | 0.5 | C1339 | 2.5 |
| C13396 | 1.5 | C13397 | 0.5 | C13398 | 0.5 | C13399 | 0.5 |
| C134 | 1900 | C1340 | 50.5 | C13400 | 0.5 | C13409 | 1.5 |
| C1341 | 0.5 | C13411 | 1.5 | C13414 | 0.5 | C13417 | 1.5 |
| C13422 | 1 | C13423 | 0.5 | C13431 | 0.5 | C13433 | 1 |
| C13437 | 3 | C13439 | 0.5 | C13440 | 1 | C13443 | 1.5 |
| C13448 | 0.5 | C13449 | 0.5 | C13452 | 0.5 | C13453 | 1.5 |
| C13455 | 1.5 | C13459 | 0.5 | C13462 | 1.5 | C13464 | 0.5 |
| C13466 | 0.5 | C13468 | 0.5 | C13469 | 1.5 | C13470 | 0.5 |
| C13475 | 1.5 | C13478 | 0.5 | C13479 | 1.5 | C13480 | 0.5 |
| C13481 | 0.5 | C13483 | 0.5 | C13484 | 0.5 | C13485 | 0.5 |
| C13486 | 1 | C13489 | 0.5 | C13497 | 1.5 | C13464 | 0.5 |
| C135 | 60 | C13501 | 2.5 | C13503 | 0.5 | C1351 | 1 |
| C13504 | 0.5 | C13512 | 1.5 | C13527 | 1.5 | C13528 | 0.5 |
| C13534 | 1.5 | C13536 | 1 | C13538 | 1.5 | C13542 | 0.5 |
| C13546 | 0.5 | C13559 | 1 | C13562 | 1.5 | C13566 | 0.5 |
| C13574 | 0.5 | C13577 | 2 | C13579 | 0.5 | C13588 | 0.5 |
| C13589 | 0.5 | C1391 | 1 | C13593 | 0.5 | C13599 | 1 |
| C13600 | 0.5 | C13607 | 2.5 | C13610 | 1.5 | C13617 | 0.5 |
| C13612 | 0.5 | C1362 | 1 | C13622 | 0.5 | C13623 | 0.5 |
| C13631 | 9 | C13634 | 2.5 | C13639 | 1 | C13641 | 1 |
| C13644 | 0.5 | C13646 | 1.5 | C13652 | 0.5 | C13653 | 1 |
| C13657 | 0.5 | C13659 | 1 | C13660 | 1.5 | C13667 | 0.5 |
| C13671 | 0.5 | C13673 | 0.5 | C13674 | 0.5 | C13683 | 0.5 |
| C8010 | 3 | C801 | 2.5 | C8005 | 0.5 | C7998 | 0.5 |
| C7980 | 7.5 | C7974 | 3.5 | C7969 | 0.5 | C7966 | 0.5 |
| C7946 | 0.5 | C7944 | 0.5 | C7934 | 2.2 | C7933 | 0.5 |
| C7931 | 0.5 | C7917 | 0.5 | C7914 | 2.5 | C7913 | 0.5 |
| C791 | 0.5 | C7900 | 0.5 | C7898 | 5 | C7893 | 1 |
| C7890 | 0.5 | C7887 | 0.5 | C7884 | 1 | C788 | 20 |
| C8064 | 1.5 | C8050 | 0.5 | C8044 | 0.5 | C8032 | 0.5 |

**Table 6.** Average number $n$ of subsequent users per multi-user computer (continued)

| Computer | n | Computer | n | Computer | n | Computer | n |
|---|---|---|---|---|---|---|---|
| C14263 | 1 | C14267 | 1 | C14268 | 0.5 | C1427 | 1 |
| C14277 | 0.5 | C14283 | 0.5 | C14290 | 0.5 | C14291 | 0.5 |
| C14299 | 1 | C14309 | 0.5 | C14322 | 1 | C14328 | 1.5 |
| C14357 | 0.5 | C14361 | 0.5 | C14163 | 0.5 | C14364 | 1 |
| C14372 | 1.5 | C14374 | 390 | C14375 | 0.5 | C14377 | 0.5 |
| C14378 | 0.5 | C14379 | 0.5 | C14380 | 0.5 | C14382 | 1 |
| C14382 | 1 | C14387 | 0.5 | C14389 | 1 | C14390 | 1 |
| C144 | 1214 | C14404 | 1 | C14416 | 0.5 | C14419 | 1 |
| C14427 | 1 | C14442 | 0.5 | C14445 | 0.5 | C14453 | 0.5 |
| C14458 | 0.5 | C14460 | 0.5 | C14464 | 0.5 | C14489 | 1 |
| C145 | 847 | C1450 | 5.5 | C14503 | 0.5 | C14509 | 0.5 |
| C14511 | 0.5 | C14512 | 0.5 | C14515 | 1 | C14519 | 1 |
| C14523 | 1.5 | C14530 | 0.5 | C14538 | 1 | C14540 | 0.5 |
| C14549 | 0.5 | C14555 | 0.5 | C14560 | 1 | C14565 | 1 |
| C14569 | 1 | C14580 | 0.5 | C14588 | 0.5 | C14594 | 0.5 |
| C146 | 947 | C14603 | 0.5 | C14608 | 1 | C14609 | 0.5 |
| C14617 | 0.5 | C14633 | 0.5 | C14637 | 0.5 | C14639 | 0.5 |
| C14640 | 0.5 | C14657 | 0.5 | C14666 | 0.5 | C14667 | 0.5 |
| C14675 | 0.5 | C14676 | 0.5 | C14682 | 0.5 | C14686 | 0.5 |
| C14693 | 0.5 | C14700 | 1 | C14711 | 1 | C14713 | 0.5 |
| C1472 | 0.5 | C14721 | 1 | C14728 | 0.5 | C14729 | 0.5 |
| C1474 | 22.5 | C1475 | 26 | C14759 | 0.5 | C14770 | 0.5 |
| C148 | 277 | C14805 | 1 | C1498 | 10.5 | C1499 | 0.5 |
| C150 | 370 | C151 | 2453 | C1512 | 2.5 | C1516 | 2 |
| C1520 | 0.5 | C1524 | 0.5 | C153 | 9 | C154 | 11.5 |
| C1541 | 17.6 | C1543 | 2.5 | C1547 | 9.5 | C1548 | 0.5 |
| C155 | 11 | C1557 | 0.5 | C1558 | 0.5 | C1560 | 1 |
| C1562 | 0.5 | C157 | 87 | C1572 | 22.4 | C1588 | 0.5 |
| C160 | 7.6 | C1602 | 1 | C1606 | 1 | C1607 | 4.5 |
| C14156 | 0.5 | C14158 | 1.5 | C14184 | 0.5 | C14202 | 0.5 |
| C14210 | 1.5 | C14212 | 1.5 | C14216 | 0.5 | C14232 | 1.5 |
| C14234 | 0.5 | C14243 | 0.5 | C1425 | 59 | C14250 | 0.5 |
| C6288 | 1 | C6292 | 5.8 | C630 | 11.6 | C6303 | 1 |
| C6304 | 2.5 | C6305 | 4 | C6308 | 1.4 | C6309 | 0.5 |
| C6314 | 3 | C6315 | 1.5 | C6318 | 1.5 | C6323 | 5 |
| C10820 | 1 | C10822 | 1 | C10823 | 1 | C11267 | 1 |
| C8027 | 0.5 | C8017 | 4.5 | C8014 | 2.5 | C8012 | 0.5 |

**Table 6.** Average number $n$ of subsequent users per multi-user computer (continued)

| Computer | n | Computer | n | Computer | n | Computer | n |
|---|---|---|---|---|---|---|---|
| C2126 | 0.5 | C213 | 54.5 | C2135 | 0.5 | C2136 | 1 |
| C2139 | 1 | C215 | 2 | C2158 | 1.5 | C2168 | 0.5 |
| C2169 | 0.5 | C2176 | 2 | C2178 | 0.5 | C2181 | 6 |
| C2182 | 0.5 | C2185 | 0.5 | C2188 | 0.5 | C219 | 312 |
| C2191 | 0.5 | C2197 | 0.5 | C2199 | 5 | C2207 | 4.5 |
| C221 | 10 | C2212 | 0.5 | C2217 | 1 | C2218 | 66.5 |
| C2219 | 0.5 | C2221 | 0.5 | C2223 | 0.5 | C2229 | 5.5 |
| C2231 | 0.5 | C2235 | 0.5 | C2240 | 3.5 | C2247 | 4.5 |
| C225 | 189 | C2254 | 48.5 | C2258 | 1 | C2260 | 1.5 |
| C2262 | 1 | C2265 | 1.5 | C2275 | 0.5 | C2279 | 2 |
| C228 | 4 | C2281 | 22.5 | C2295 | 5 | C2289 | 1.5 |
| C2300 | 2.5 | C2303 | 2 | C231 | 200 | C2313 | 0.5 |
| C2314 | 3.6 | C2317 | 1.5 | C2320 | 0.5 | C2328 | 1.5 |
| C234 | 285 | C2340 | 1 | C2343 | 1.5 | C2352 | 24.5 |
| C2359 | 74 | C236 | 1 | C2367 | 0.5 | C2367 | 0.5 |
| C237 | 285 | C2370 | 0.5 | C2371 | 0.5 | C2372 | 1 |
| C2375 | 0.5 | C2377 | 7 | C238 | 3.5 | C2382 | 0.5 |
| C2387 | 0.5 | C2388 | 4 | C239 | 23.6 | C2392 | 0.5 |
| C2393 | 0.5 | C2394 | 259 | C2395 | 6.5 | C2405 | 0.5 |
| C2409 | 0.5 | C2411 | 1 | C2412 | 0.5 | C2416 | 0.5 |
| C2430 | 23.5 | C2432 | 1 | C2433 | 1 | C244 | 1.5 |
| C2442 | 4 | C245 | 1 | C2447 | 1 | C2451 | 0.5 |
| C2460 | 86.5 | C2467 | 1.5 | C247 | 0.5 | C2472 | 78.5 |
| C248 | 314 | C2487 | 2 | C2488 | 0.5 | C2489 | 19.5 |
| C249 | 0.5 | C2492 | 0.5 | C2494 | 5 | C2497 | 0.5 |
| C251 | 492 | C2512 | 0.5 | C2517 | 0.5 | C252 | 1 |
| C2530 | 2 | C2533 | 1 | C2538 | 1.5 | C254 | 1 |
| C261 | 204 | C2610 | 1.5 | C2617 | 0.5 | C263 | 11 |
| C264 | 6.6 | C2642 | 8 | C265 | 2.8 | C2652 | 1 |
| C266 | 2.5 | C266 | 3 | C2660 | 0.5 | C2668 | 2 |
| C2669 | 1.5 | C2670 | 0.5 | C2675 | 8.5 | C268 | 284 |
| C270 | 25.5 | C2701 | 25.02 | C2704 | 2.5 | C2718 | 13.5 |
| C2722 | 0.5 | C2725 | 2 | C2726 | 1 | C2747 | 0.5 |
| C2764 | 7 | C2765 | 2 | C2726 | 6 | C2779 | 10.5 |
| C2787 | 1 | C2792 | 1.5 | C2793 | 8 | C2794 | 7 |
| C280 | 34.5 | C281 | 7 | C2810 | 39.5 | C2818 | 13.52 |
| C284 | 11 | C2862 | 12.5 | C2891 | 6 | C2899 | 4 |

**Table 6.** Average number $n$ of subsequent users per multi-user computer (continued)

| Computer | n | Computer | n | Computer | n | Computer | n |
|---|---|---|---|---|---|---|---|
| C3532 | 0.5 | C354 | 1 | C3542 | 0.5 | C355 | 0.5 |
| C3553 | 1.5 | C3556 | 1 | C3558 | 0.5 | C3561 | 1 |
| C3577 | 0.5 | C3482 | 0.5 | C3586 | 1.5 | C3592 | 2 |
| C3607 | 1 | C3610 | 0.5 | C3618 | 25 | C3621 | 5.5 |
| C3623 | 0.5 | C3632 | 0.5 | C3649 | 1 | C3652 | 0.5 |
| C3658 | 1.5 | C3663 | 340 | C3667 | 0.5 | C3677 | 1 |
| C3694 | 1.5 | C371 | 1 | C372 | 6.5 | C3725 | 1 |
| C3726 | 5 | C3750 | 7 | C3758 | 199 | C3760 | 3.5 |
| C3772 | 8 | C3776 | 5.5 | C3779 | 1 | C378 | 0.5 |
| C3816 | 20.5 | C3825 | 1 | C3840 | 0.5 | C3843 | 1.5 |
| C3849 | 11.5 | C3850 | 7.6 | C3851 | 10 | C3861 | 1 |
| C3867 | 1.5 | C3870 | 1 | C3872 | 5.5 | C3873 | 6 |
| C388 | 4 | C3882 | 8 | C3884 | 2.5 | C3890 | 0.5 |
| C3891 | 1.5 | C3903 | 0.5 | C3914 | 2.5 | C3915 | 32.9 |
| C3918 | 1.5 | C3935 | 0.5 | C394 | 38 | C3944 | 318 |
| C3962 | 7.5 | C3981 | 26.5 | C3985 | 14 | C3915 | 32.9 |
| C3987 | 27.2 | C3994 | 1.5 | C4 | 1 | C4008 | 6 |
| C4009 | 8 | C4010 | 8 | C4014 | 1 | C4016 | 5.8 |
| C4019 | 7.5 | C4035 | 9 | C4040 | 1.5 | C405 | 20 |
| C4052 | 1.5 | C4058 | 2.4 | C4060 | 0.5 | C4070 | 0.5 |
| C4073 | 1.5 | C409 | 5.5 | C4060 | 0.5 | C4094 | 1.5 |
| C411 | 0.5 | C4115 | 15 | C4116 | 0.5 | C4122 | 230 |
| C4128 | 300 | C414 | 84.5 | C415 | 143 | C4160 | 2 |
| C4162 | 1 | C4166 | 1 | C417 | 1 | C4185 | 1.5 |
| C4192 | 1 | C42 | 0.5 | C4200 | 0.5 | C4217 | 1 |
| C4227 | 1 | C4241 | 1 | C4241 | 1 | C425 | 37.5 |
| C4250 | 1.5 | C4254 | 1.5 | C4258 | 19.5 | C4259 | 18 |
| C4260 | 10 | C4263 | 1 | C4266 | 24.5 | C4270 | 9 |
| C427 | 1.8 | C4270 | 8 | C4271 | 8.5 | C428 | 8 |
| C4280 | 2 | C429 | 1 | C4293 | 39.5 | C4299 | 1 |
| C4320 | 1.5 | C4323 | 1 | C4271 | 3.5 | C4364 | 1 |
| C4373 | 1 | C4374 | 1.5 | C4378 | 1.5 | C4381 | 2.6 |
| C4390 | 0.5 | C4394 | 4.5 | C440 | 29 | C4408 | 8.5 |
| C4417 | 0.5 | C4422 | 5 | C4429 | 2 | C443 | 648 |
| C4431 | 1 | C4434 | 1.5 | C4437 | 1 | C4442 | 1.5 |
| C4445 | 1.5 | C4447 | 3.4 | C4450 | 58 | C4457 | 0.5 |
| C4458 | 0.5 | C4465 | 7.5 | C4466 | 28.5 | C4469 | 1.5 |

**Table 6.** Average number $n$ of subsequent users per multi-user computer (continued)

| Computer | n | Computer | n | Computer | n | Computer | n |
|---|---|---|---|---|---|---|---|
| C4782 | 14.7 | C4784 | 232 | C4785 | 412 | C4787 | 4.5 |
| C4790 | 4 | C4791 | 4.5 | C4795 | 9.5 | C4796 | 290 |
| C4803 | 13.1 | C4804 | 3.5 | C4805 | 7.5 | C4814 | 5 |
| C4815 | 23.5 | C4818 | 5.5 | C4824 | 6 | C4826 | 8 |
| C4834 | 11.7 | C4835 | 19.5 | C4838 | 59 | C4859 | 16.4 |
| C486 | 4 | C4863 | 33 | C4877 | 23.5 | C4880 | 28.5 |
| C4896 | 43 | C4904 | 16.24 | C4907 | 6.6 | C4911 | 31.5 |
| C4920 | 3.4 | C4930 | 5.5 | C4932 | 15 | C4933 | 1 |
| C4936 | 8.5 | C4949 | 5 | C4955 | 7.5 | C4956 | 23.1 |
| C5010 | 1 | C5022 | 2.5 | C5035 | 41 | C5039 | 7.5 |
| C5046 | 9.7 | C5047 | 7.6 | C5064 | 4.7 | C5071 | 4.5 |
| C508 | 13.6 | C5088 | 4.4 | C51 | 5 | C5115 | 24.5 |
| C5128 | 27.2 | C5134 | 5.5 | C517 | 22.4 | C5170 | 6 |
| C5194 | 7.8 | C5200 | 9.4 | C5214 | 6 | C5251 | 2.7 |
| C5284 | 5 | C5286 | 50.2 | C5288 | 2.1 | C5290 | 8 |
| C53 | 455 | C534 | 7 | C5351 | 0.5 | C5358 | 1.5 |
| C5403 | 1.5 | C5405 | 2 | C5423 | 48.4 | C5435 | 17.6 |
| C5449 | 23.7 | C5453 | 1 | C5468 | 33.4 | C5481 | 22.5 |
| C5490 | 12 | C5500 | 2 | C5513 | 228 | C5537 | 11.5 |
| C5553 | 450 | C5564 | 13 | C5578 | 12.75 | C5581 | 5.5 |
| C5602 | 4 | C5617 | 4.5 | C5619 | 1 | C5617 | 4.8 |
| C5629 | 15 | C5639 | 1 | C5651 | 1 | C5658 | 4 |
| C5659 | 18 | C5661 | 3 | C568 | 20.1 | C5683 | 5 |
| C5702 | 24.1 | C5712 | 2.9 | C5752 | 285 | C576 | 1.5 |
| C5770 | 1.5 | C5787 | 3.4 | C5810 | 6.46 | C5818 | 1.8 |
| C582 | 0.5 | C5837 | 0.5 | C5844 | 0.5 | C5850 | 0.5 |
| C5862 | 1.5 | C5881 | 1.5 | C5897 | 1 | C5900 | 1 |
| C5988 | 6.5 | C5992 | 1 | C5996 | 0.5 | C6000 | 0.5 |
| C6007 | 37.5 | C6016 | 1.5 | C602 | 19 | C6020 | 24.6 |
| C6040 | 2 | C6044 | 3 | C6053 | 1 | C6068 | 1.5 |
| C6107 | 1 | C6111 | 1 | C6113 | 7.5 | C6119 | 1 |
| C6122 | 0.5 | C6130 | 0.5 | C6141 | 1.5 | C6146 | 2.8 |
| C6153 | 6.5 | C6160 | 345 | C6165 | 1.5 | C6168 | 29.5 |
| C6171 | 7.5 | C6173 | 2 | C6181 | 1.5 | C6190 | 2.5 |
| C6194 | 0.5 | C6200 | 1 | C6204 | 1.5 | C6206 | 0.5 |
| C6220 | 1.5 | C6226 | 1.5 | C6236 | 8.6 | C6242 | 1.5 |
| C6249 | 4 | C6262 | 1.5 | C6271 | 0.5 | C6281 | 4.5 |

**Table 6.** Average number $n$ of subsequent users per multi-user computer (continued)

| Computer | n | Computer | n | Computer | n | Computer | n |
|---|---|---|---|---|---|---|---|
| C6887 | 1.5 | C6890 | 0.5 | C6898 | 0.5 | C6901 | 0.5 |
| C6910 | 0.5 | C6912 | 1 | C6918 | 0.5 | C6921 | 2 |
| C6925 | 0.5 | C6926 | 0.5 | C6930 | 5 | C6931 | 0.5 |
| C6933 | 0.5 | C6938 | 1 | C694 | 5.5 | C6942 | 0.5 |
| C6956 | 0.5 | C6959 | 0.5 | C6956 | 0.5 | C6959 | 0.5 |
| C6962 | 1.5 | C6966 | 0.5 | C6969 | 1 | C6971 | 4.5 |
| C6977 | 12.5 | C6979 | 0.5 | C6987 | 1 | C6988 | 2.5 |
| C6995 | 1 | C6998 | 17.4 | C70 | 0.5 | C7001 | 2 |
| C7010 | 1 | C7012 | 1.5 | C7014 | 0.5 | C7022 | 0.5 |
| C7029 | 3.2 | C703 | 2 | C7032 | 0.5 | C7042 | 0.5 |
| C7043 | 0.5 | C7048 | 1 | C7057 | 4.5 | C7060 | 0.5 |
| C7066 | 1.5 | C7075 | 1 | C7081 | 0.5 | C7083 | 4 |
| C7084 | 14.6 | C7090 | 2.5 | C7091 | 2.4 | C7102 | 0.5 |
| C7133 | 3 | C7134 | 2 | C7151 | 1.5 | C7152 | 25 |
| C716 | 1 | C7164 | 3.5 | C7175 | 2.5 | C7190 | 0.5 |
| C720 | 830 | C7212 | 1.5 | C7222 | 0.5 | C7226 | 0.5 |
| C7229 | 1 | C7231 | 1 | C7232 | 4.3 | C7244 | 1.5 |
| C7252 | 0.5 | C7256 | 2.6 | C7262 | 0.5 | C7266 | 5 |
| C7273 | 4 | C7278 | 0.5 | C7291 | 3 | C7307 | 2.5 |
| C7313 | 1 | C7329 | 1.5 | C734 | 2.5 | C7356 | 0.5 |
| C7357 | 0.5 | C7358 | 0.5 | C7360 | 3.6 | C7364 | 26 |
| C7376 | 4 | C7380 | 1 | C7386 | 0.5 | C7392 | 1.5 |
| C7394 | 1.5 | C74 | 1.6 | C7403 | 0.5 | C7404 | 24 |
| C7420 | 1 | C7422 | 6.5 | C7423 | 3.5 | C7424 | 1 |
| C7425 | 2.5 | C7429 | 0.5 | C7445 | 0.5 | C7446 | 1 |
| C7447 | 1.5 | C7451 | 1 | C7455 | 0.5 | C7463 | 0.5 |
| C7472 | 1 | C748 | 37.5 | C749 | 1.5 | C75 | 9.5 |
| C7515 | 0.5 | C7528 | 3 | C7533 | 0.5 | C7541 | 3 |
| C7553 | 0.5 | C7557 | 1.5 | C7574 | 1 | C7599 | 0.5 |
| C7607 | 1 | C7609 | 1 | C7618 | 1 | C7623 | 1 |
| C7633 | 0.5 | C7640 | 0.5 | C7648 | 2 | C7650 | 3 |
| C7651 | 0.5 | C7654 | 0.5 | C7656 | 1.5 | C766 | 4.5 |
| C7660 | 4 | C7673 | 2.8 | C7681 | 0.5 | C769 | 13 |
| C7691 | 4.5 | C770 | 0.5 | C7714 | 1.5 | C7719 | 0.5 |
| C7722 | 5 | C7725 | 5 | C7726 | 2 | C7728 | 0.5 |
| C7737 | 1 | C7741 | 2 | C7742 | 3 | C775 | 87 |
| C7750 | 1 | C7752 | 0.5 | C7755 | 0.5 | C7757 | 0.5 |

**Table 6.** Average number $n$ of subsequent users per multi-user computer (continued)

| Computer | n | Computer | n | Computer | n | Computer | n |
|---|---|---|---|---|---|---|---|
| C209 | 37.5 | C2098 | 10 | C21 | 1 | C210 | 2.3 |
| C2107 | 1.5 | C211 | 2 | C2110 | 1.5 | C2115 | 1.7 |
| C3476 | 2.8 | C3477 | 0.5 | C3485 | 0.5 | C3488 | 1.5 |
| C3490 | 11 | C3494 | 1.5 | C3499 | 6.5 | C351 | 1.5 |
| C3518 | 7.5 | C3452 | 0.5 | C3521 | 2.5 | C3529 | 9 |
| C4748 | 30 | C4752 | 4 | C4755 | 3.4 | C4758 | 3 |
| C476 | 853 | C4760 | 1 | C4761 | 8 | C4762 | 3 |
| C4767 | 38.5 | C4769 | 6.5 | C4779 | 25.5 | C4781 | 7 |
| C6802 | 1.5 | C6812 | 0.5 | C6821 | 3.5 | C6825 | 3.5 |
| C6827 | 40 | C6831 | 2.5 | C6842 | 0.5 | C6843 | 0.5 |
| C6848 | 1 | C6858 | 0.5 | C6867 | 3 | C6880 | 0.5 |
| C10010 | 0.5 | C10011 | 0.5 | C10012 | 0.5 | C10019 | 1 |
| C10031 | 0.6 | C10037 | 2 | C10041 | 2 | C10043 | 1 |
| C10047 | 2.75 | C10003 | 3 | C10006 | 7.5 | C10052 | 1 |
| C1134 | 2.87 | C10052 | 2 | C10003 | 3 | C10046 | 2.5 |
| C11030 | 0.5 | C11031 | 0.5 | C11034 | 0.5 | C11432 | 1 |
| C11040 | 0.5 | C11041 | 0.5 | C11043 | 0.5 | C11433 | 0.5 |
| C11044 | 0.5 | C11045 | 0.5 | C11049 | 1.5 | C11435 | 0.5 |
| C11051 | 0.5 | C11054 | 0.5 | C11057 | 1 | C11439 | 0.5 |
| C1242 | 0.5 | C12423 | 80.5 | C12425 | 2.5 | C12428 | 0.5 |
| C12432 | 0.5 | C12438 | 1 | C1244 | 1 | C12446 | 1.5 |
| C12463 | 1.5 | C12464 | 1 | C12470 | 1 | C12474 | 1.5 |
| C12478 | 1.5 | C12479 | 3.2 | C12480 | 0.5 | C12484 | 0.5 |
| C12486 | 1.5 | C12489 | 1.5 | C12490 | 0.5 | C12493 | 1 |
| C12492 | 1 | C12497 | 0.5 | C12499 | 2 | C12499 | 2.5 |
| C125 | 1.5 | C1250 | 1.5 | C12506 | 0.5 | C12513 | 0.5 |
| C12520 | 0.5 | C12525 | 0.5 | C124526 | 0.5 | C1254 | 6.5 |
| C12540 | 1 | C12541 | 0.5 | C12543 | 0.5 | C12548 | 1.8 |
| C12551 | 0.5 | C12555 | 1.5 | C12556 | 0.5 | C12558 | 1.5 |
| C1256 | 0.5 | C12563 | 0.5 | C12566 | 2 | C1257 | 0.5 |
| C12578 | 8 | C12583 | 0.5 | C12589 | 1.5 | C12593 | 0.5 |
| C126 | 1.5 | C12600 | 0.5 | C12601 | 1.5 | C12603 | 1 |
| C12604 | 0.5 | C12608 | 1.5 | C12609 | 2 | C12610 | 1.5 |
| C12611 | 0.5 | C12616 | 6.4 | C12619 | 0.5 | C12621 | 0.8 |
| C12625 | 0.5 | C1263 | 1 | C12630 | 0.5 | C12626 | 0.5 |
| C12628 | 0.5 | C12632 | 1 | C12634 | 1.5 | C12639 | 1.5 |
| C12640 | 8.1 | C12641 | 1.5 | C12644 | 1 | C12651 | 0.5 |

**Table 6.** Average number $n$ of subsequent users per multi-user computer (continued)

| Computer | n | Computer | n | Computer | n | Computer | n |
|---|---|---|---|---|---|---|---|
| C10725 | 0.6 | C10729 | 0.5 | C1073 | 0.6 | C11237 | 2.5 |
| C10731 | 0.5 | C10733 | 0.5 | C10737 | 1 | C11239 | 2 |
| C10738 | 1 | C10739 | 1 | C10741 | 0.5 | C11242 | 1 |
| C10742 | 1.5 | C10744 | 0.5 | C10745 | 0.5 | C11244 | 1 |
| C10746 | 4 | C10747 | 0.5 | C10750 | 0.5 | C11246 | 1 |
| C10753 | 1.5 | C10759 | 1 | C10760 | 0.5 | C11247 | 1.5 |
| C10761 | 1 | C10763 | 2 | C10764 | 1.5 | C11248 | 1 |
| C10767 | 1 | C10768 | 1 | C1077 | 1 | C11250 | 0.5 |
| C10770 | 1 | C10776 | 0.5 | C10777 | 0.5 | C1125 | 0.5 |
| C10778 | 0.5 | C10781 | 0.5 | C10783 | 0.5 | C11252 | 1 |
| C10786 | 1 | C10787 | 1 | C10789 | 0.5 | C11254 | 0.5 |
| C1079 | 5.5 | C10791 | 1.5 | C10796 | 0.5 | C11255 | 2 |
| C10796 | 0.5 | C108 | 22.4 | C10800 | 1 | C11257 | 0.5 |
| C10804 | 1 | C10807 | 0.5 | C10810 | 2 | C11260 | 3.5 |
| C106 | 2.5 | C1060 | 4.3 | C10601 | 2.6 | C11347 | 0.5 |
| C10605 | 2 | C10608 | 0.5 | C10609 | 2 | C11348 | 1 |
| C10617 | 0.5 | C10621 | 0.5 | C10622 | 3 | C11349 | 1.5 |
| C10624 | 0.5 | C10627 | 0.5 | C10632 | 0.5 | C1135 | 17.5 |
| C10634 | 1 | C10635 | 1 | C10636 | 0.5 | C11350 | 1.5 |
| C10639 | 3 | C10640 | 0.5 | C10643 | 2.6 | C11351 | 0.5 |
| C10644 | 2 | C10646 | 0.5 | C10648 | 1 | C11354 | 1 |
| C10650 | 0.5 | C10651 | 1 | C10652 | 1 | C11355 | 2.5 |
| C10654 | 1.6 | C10655 | 1 | C10656 | 0.5 | C11357 | 1.5 |
| C10657 | 1 | C10660 | 0.5 | C10661 | 0.5 | C11358 | 0.5 |
| C10666 | 0.5 | C10669 | 1 | C10670 | 2.8 | C11359 | 0.5 |
| C10672 | 0.5 | C10674 | 2 | C10676 | 0.5 | C1136 | 0.5 |
| C10678 | 0.5 | C10679 | 1 | C10680 | 0.5 | C11361 | 0.5 |
| C10683 | 0.5 | C10684 | 0.5 | C10685 | 0.5 | C11367 | 1.7 |
| C10686 | 0.5 | C10688 | 1 | C10689 | 2.6 | C1137 | 0.5 |
| C107 | 60.5 | C10700 | 0.5 | C10701 | 1.5 | C11372 | 0.5 |
| C10703 | 1 | C10705 | 1.6 | C10708 | 0.5 | C11376 | 2.5 |
| C10710 | 1.6 | C10713 | 0.5 | C10715 | 1.5 | C11378 | 0.5 |
| C10712 | 0.5 | C10716 | 0.5 | C10717 | 0.5 | C11380 | 1 |
| C10719 | 0.5 | C10720 | 0.5 | C10724 | 0.6 | C11383 | 1 |
| C10824 | 2 | C10826 | 0.5 | C10829 | 0.5 | C11269 | 0.5 |
| C12685 | 1.5 | C12688 | 0.5 | C1269 | 0.5 | C12693 | 1 |
| C12694 | 0.5 | C12696 | 1 | C127 | 4.5 | C12701 | 0.5 |

**Table 6.** Average number $n$ of subsequent users per multi-user computer (continued)

| Computer | n | Computer | n | Computer | n | Computer | n |
|---|---|---|---|---|---|---|---|
| C11058 | 5 | C11059 | 4.5 | C11060 | 2.5 | C11440 | 0.5 |
| C11062 | 4 | C11063 | 2.5 | C11064 | 5 | C11446 | 0.5 |
| C11065 | 3.5 | C11066 | 3.5 | C11067 | 6 | C11448 | 0.5 |
| C11068 | 3.5 | C11066 | 3.5 | C11067 | 11.5 | C11450 | 0.5 |
| C1107 | 0.5 | C11070 | 1 | C11072 | 1.7 | C11452 | 1 |
| C11074 | 4.5 | C11075 | 3.5 | C11077 | 0.5 | C11453 | 0.5 |
| C11079 | 5 | C11081 | 2 | C1108 | 1 | C11458 | 1 |
| C11082 | 2.5 | C11085 | 0.5 | C11086 | 2 | C11462 | 1 |
| C11087 | 1 | C11093 | 0.5 | C11097 | 1 | C11463 | 2 |
| C111 | 5.2 | C11101 | 0.5 | C11102 | 2.3 | C11468 | 0.5 |
| C11106 | 1 | C1111 | 16 | C11110 | 0.5 | C1147 | 1.5 |
| C11111 | 1 | C11112 | 3 | C11113 | 2.5 | C11470 | 0.5 |
| C11118 | 0.5 | C11121 | 1 | C11122 | 2 | C11471 | 0.5 |
| C11123 | 1 | C11126 | 0.5 | C11128 | 2.5 | C11472 | 1 |
| C11131 | 0.5 | C11132 | 0.5 | C11133 | 0.5 | C11474 | 0.5 |
| C111345 | 0.5 | C11139 | 0.5 | C1114 | 0.5 | C11475 | 1.5 |
| C11141 | 0.5 | C11142 | 0.5 | C1115 | 0.5 | C11477 | 0.5 |
| C11150 | 2.5 | C11152 | 2 | C11154 | 0.5 | C11483 | 0.5 |
| C1083 | 173.5 | C10830 | 1 | C10832 | 3 | C11270 | 1.5 |
| C11840 | 2 | C11842 | 2.8 | C11848 | 2.8 | C11849 | 0.5 |
| C11851 | 0.5 | C11853 | 2.5 | C11857 | 1 | C11859 | 0.5 |
| C117860 | 1 | C11861 | 1 | C11872 | 0.5 | C11880 | 0.5 |
| C11882 | 3.5 | C11886 | 0.5 | C11888 | 0.5 | C11893 | 2 |
| C11896 | 0.5 | C11899 | 0.5 | C11902 | 1 | C11906 | 0.5 |
| C11909 | 1 | C11910 | 0.5 | C11911 | 0.5 | C11913 | 0.5 |
| C11914 | 1 | C11920 | 2 | C11921 | 1 | C11922 | 0.5 |
| C11923 | 1 | C11928 | 1 | C1193 | 0.5 | C11937 | 1 |
| C11941 | 2 | C11942 | 0.5 | C11943 | 1 | C11944 | 0.5 |
| C11946 | 0.5 | C1195 | 0.5 | C11793 | 1 | C11952 | 2 |
| C11955 | 0.5 | C11956 | 0.5 | C11960 | 1 | C11961 | 1 |
| C11962 | 0.5 | C11963 | 1 | C11964 | 0.5 | C11971 | 0.5 |
| C11973 | 0.5 | C11975 | 0.5 | C11976 | 2.5 | C11983 | 2.5 |
| C11984 | 1 | C11987 | 0.5 | C11988 | 1 | C11990 | 0.5 |
| C11993 | 1 | C11997 | 0.5 | C11999 | 2.4 | C11794 | 1 |
| C120 | 32.8 | C1200 | 1 | C12000 | 0.5 | C12005 | 0.5 |
| C12007 | 0.5 | C12011 | 2.5 | C12012 | 1.5 | C12013 | 1.5 |
| C12014 | 1 | C12016 | 0.5 | C12022 | 5.5 | C12023 | 3 |

**Table 6.** Average number $n$ of subsequent users per multi-user computer (continued)

| Computer | n | Computer | n | Computer | n | Computer | n |
|---|---|---|---|---|---|---|---|
| C12991 | 0.5 | C12994 | 1 | C12997 | 1.5 | C12999 | 1 |
| C13 | 1 | C130 | 8.5 | C13002 | 0.5 | C13005 | 1 |
| C13008 | 0.5 | C13010 | 0.5 | C13012 | 0.5 | C13015 | 0.5 |
| C13016 | 1.5 | C13021 | 0.5 | C13024 | 0.5 | C13027 | 2 |
| C13034 | 0.5 | C13044 | 0.5 | C13046 | 0.5 | C13052 | 0.5 |
| C13053 | 0.5 | C13056 | 0.5 | C13058 | 0.5 | C13060 | 0.5 |
| C13061 | 0.5 | C13062 | 0.5 | C13065 | 0.5 | C13068 | 0.5 |
| C13070 | 0.5 | C13071 | 0.5 | C13073 | 0.5 | C13077 | 0.5 |
| C13082 | 1 | C13083 | 0.5 | C13086 | 0.5 | C13087 | 0.5 |
| C13088 | 1 | C13089 | 0.5 | C13090 | 0.5 | C13091 | 0.5 |
| C13094 | 1.5 | C13097 | 0.5 | C13098 | 0.5 | C13099 | 1 |
| C131 | 2.5 | C13110 | 0.5 | C130101 | 0.5 | C13010 | 1.5 |
| C13107 | 2 | C13108 | 1 | C1311 | 9.5 | C13110 | 0.5 |
| C13143 | 1 | C13137 | 0.5 | C13121 | 1 | C13146 | 0.5 |
| C13150 | 1 | C13153 | 0.5 | C13156 | 0.5 | C13164 | 0.5 |
| C13165 | 1.5 | C13169 | 0.5 | C13172 | 0.5 | C13177 | 1 |
| C13181 | 2 | C13183 | 2.5 | C13187 | 0.5 | C13189 | 0.5 |
| C13193 | 1 | C13195 | 0.5 | C13196 | 0.5 | C132 | 27 |
| C13202 | 1.5 | C13204 | 0.5 | C13207 | 0.5 | C13214 | 0.5 |
| C13219 | 0.5 | C1322 | 14.5 | C13232 | 1 | C13234 | 0.5 |
| C13232 | 1 | C13234 | 0.5 | C13236 | 0.5 | C13237 | 1 |
| C13242 | 1 | C13244 | 1 | C13246 | 0.5 | C13249 | 1 |
| C13252 | 0.5 | C13253 | 0.5 | C13255 | 0.5 | C13256 | 1 |
| C8950 | 8 | C8949 | 7 | C89 | 11 | C886 | 116 |
| C8847 | 5.5 | C8816 | 6 | C88 | 8 | C8761 | 7 |
| C875 | 11.5 | C8740 | 5 | C87 | 4.5 | C8684 | 8 |
| C8664 | 3.9 | C8618 | 7.5 | C8600 | 3.5 | C86 | 4.5 |
| C8596 | 94 | C8576 | 10 | C8539 | 15 | C8530 | 6.5 |
| C8521 | 7 | C850 | 144 | C9521 | 4.2 | C9518 | 45.5 |
| C8485 | 9 | C846 | 1.5 | C8458 | 7.5 | C8449 | 2.5 |
| C8391 | 5.5 | C8390 | 4 | C8372 | 19 | C8369 | 2 |
| C8331 | 4.5 | C83 | 1100 | C8299 | 26 | C8274 | 8 |
| C8254 | 0.5 | C8246 | 0.5 | C8244 | 0.5 | C8238 | 0.5 |
| C8231 | 0.5 | C8222 | 0.5 | C8220 | 0.5 | C8219 | 0.5 |
| C8217 | 0.5 | C8210 | 1 | C8204 | 0.5 | C8200 | 0.5 |
| C821 | 1120 | C8199 | 4 | C8197 | 0.5 | C8176 | 1.5 |
| C8170 | 0.5 | C81430 | 1.5 | C8113 | 4.5 | C811 | 1 |

**Table 6.** Average number $n$ of subsequent users per multi-user computer (continued)

| Computer | n | Computer | n | Computer | n | Computer | n |
|---|---|---|---|---|---|---|---|
| C13684 | 1.5 | C13688 | 0.5 | C13689 | 0.5 | C13692 | 1 |
| C13695 | 0.5 | C13696 | 1 | C13699 | 0.5 | C137 | 19 |
| C13707 | 0.5 | C13708 | 0.5 | C13709 | 2 | C13711 | 1.5 |
| C13712 | 1 | C13717 | 1 | C13718 | 1 | C13719 | 0.5 |
| C13720 | 0.5 | C13723 | 0.5 | C13724 | 0.5 | C13728 | 1.7 |
| C13729 | 1 | C13736 | 1 | C13753 | 3.5 | C13756 | 1.5 |
| C13757 | 0.5 | C13763 | 1 | C13765 | 0.5 | C13769 | 1 |
| C13770 | 1 | C13774 | 0.5 | C13775 | 1.5 | C13777 | 1.5 |
| C13779 | 0.5 | C13780 | 1 | C13784 | 1.5 | C13788 | 0.5 |
| C13792 | 1 | C13796 | 0.5 | C13799 | 0.5 | C13800 | 0.5 |
| C13803 | 1.5 | C13804 | 0.5 | C13809 | 0.5 | C13812 | 1.5 |
| C13813 | 0.5 | C13820 | 0.5 | C13828 | 1 | C13830 | 1.5 |
| C13833 | 1 | C13849 | 1.5 | C13853 | 0.5 | C13864 | 1 |
| C13867 | 0.5 | C13870 | 0.5 | C13874 | 1 | C13881 | 1 |
| C13882 | 1 | C13891 | 0.5 | C13894 | 0.5 | C13902 | 0.5 |
| C13903 | 1.5 | C13906 | 2 | C1391 | 1 | C13927 | 1.5 |
| C13929 | 0.5 | C13937 | 1 | C13944 | 2.5 | C13950 | 0.5 |
| C13957 | 1 | C13963 | 0.5 | C13967 | 1 | C13969 | 4 |
| C13971 | 1 | C13977 | 0.5 | C13981 | 1.5 | C13984 | 0.5 |
| C13986 | 1 | C13995 | 3 | C140 | 2.5 | C14000 | 1 |
| C14002 | 1.5 | C1401 | 19 | C14010 | 0.5 | C14011 | 0.5 |
| C14130 | 1 | C14134 | 2 | C14137 | 1 | C14142 | 1 |
| C14145 | 0.5 | C14146 | 1.5 | C14150 | 1 | C14152 | 3 |
| C7762 | 0.5 | C7763 | 1 | C7766 | 0.5 | C7774 | 0.5 |
| C7781 | 3 | C7782 | 0.5 | C7787 | 1 | C7800 | 2.5 |
| C7813 | 1.5 | C783 | 38.5 | C7837 | 2 | C784 | 22.3 |
| C785 | 24.2 | C7853 | 1 | C786 | 1.5 | C7870 | 0.5 |
| C7871 | 0.5 | C7873 | 1 | C7876 | 0.5 | C7878 | 0.5 |
| C12078 | 0.5 | C12079 | 1.5 | C12080 | 1 | C12083 | 0.5 |
| C10834 | 0.5 | C10839 | 4.5 | C10840 | 0.5 | C11271 | 1 |
| C12653 | 1 | C1266 | 0.5 | C12660 | 0.5 | C12661 | 0.5 |
| C12663 | 1 | C12671 | 0.5 | C12682 | 0.5 | C12684 | 7.3 |
| C12024 | 2.7 | C12026 | 0.5 | C12043 | 6.5 | C12045 | 1 |
| C12048 | 5.4 | C12050 | 1 | C12054 | 0.5 | C12057 | 4.5 |
| C12058 | 1 | C12060 | 0.5 | C12063 | 0.5 | C12068 | 1.5 |
| C1207 | 2 | C12070 | 0.5 | C12073 | 1 | C12075 | 0.5 |

**Table 6.** Average number $n$ of subsequent users per multi-user computer (continued)

| Computer | n | Computer | n | Computer | n | Computer | n |
|---|---|---|---|---|---|---|---|
| C161 | 7.5 | C1610 | 0.5 | C1618 | 0.5 | C162 | 19.5 |
| C1622 | 1 | C1624 | 1 | C163 | 1 | C16330 | 1 |
| C1631 | 1 | C1638 | 1 | C1640 | 1 | C1643 | 1.5 |
| C1644 | 1.5 | C1652 | 0.5 | C1653 | 0.5 | C1657 | 0.5 |
| C1661 | 0.5 | C1665 | 0.5 | C1667 | 0.5 | C1670 | 0.5 |
| C1670 | 0.5 | C1676 | 0.5 | C168 | 8.5 | C1680 | 0.5 |
| C1682 | 0.5 | C1685 | 0.5 | C1689 | 96.5 | C169 | 2 |
| C1692 | 2.5 | C1694 | 99.5 | C170 | 2.5 | C172 | 0.5 |
| C1725 | 1.5 | C1734 | 5 | C1733 | 2 | C174 | 2 |
| C175 | 3 | C1767 | 32 | C177 | 752.8 | C1778 | 115 |
| C178 | 1 | C1785 | 11 | C1787 | 4.5 | C1790 | 0.5 |
| C1793 | 4.5 | C1794 | 0.5 | C1799 | 70.5 | C1801 | 0.5 |
| C1811 | 0.5 | C1815 | 0.5 | C1820 | 1 | C1826 | 0.5 |
| C1834 | 6 | C1841 | 1 | C1845 | 0.5 | C1850 | 1 |
| C1853 | 37.5 | C1857 | 1 | C1881 | 7.2 | C1883 | 173.5 |
| C1912 | 84.5 | C1919 | 5 | C192 | 24 | C1921 | 2 |
| C1931 | 1 | C1932 | 54.5 | C1942 | 3 | C1952 | 1.5 |
| C1970 | 1.5 | C1971 | 0.5 | C1972 | 2 | C1974 | 6.5 |
| C1981 | 1 | C1988 | 1 | C1991 | 1.5 | C1997 | 0.5 |
| C2 | 50 | C200 | 5000 | C2000 | 0.5 | C2004 | 3 |
| C203 | 47.5 | C2034 | 45 | C2037 | 42.5 | C204 | 39.5 |
| C2040 | 0.5 | C2041 | 1.5 | C2046 | 0.5 | C2047 | 1.5 |
| C205 | 27.9 | C2059 | 1.5 | C2064 | 44 | C2068 | 2 |
| C2074 | 2 | C2075 | 137 | C2077 | 0.5 | C2086 | 1 |
| C6333 | 25 | C6337 | 4.5 | C6340 | 0.5 | C6343 | 33 |
| C6344 | 4.5 | C6347 | 1.5 | C6357 | 1.5 | C638 | 7.6 |
| C6388 | 0.5 | C6399 | 1.5 | C6410 | 0.5 | C6424 | 0.5 |
| C6436 | 0.5 | C644 | 1.5 | C645 | 40.5 | C6457 | 0.5 |
| C6468 | 13.4 | C6474 | 0.5 | C6480 | 1.5 | C65 | 7.5 |
| C650 | 5.6 | C6500 | 1.8 | C6508 | 2.5 | C651 | 6.6 |
| C6514 | 0.5 | C6527 | 0.5 | C6531 | 0.5 | C6537 | 13.8 |
| C13259 | 1 | C13264 | 0.5 | C1327 | 1.5 | C13271 | 1 |
| C13277 | 0.5 | C13282 | 0.5 | C13284 | 0.5 | C13287 | 0.5 |
| C1329 | 6.7 | C13291 | 1 | C13293 | 0.5 | C13294 | 0.5 |
| C10812 | 9.5 | C10814 | 0.5 | C10815 | 1 | C11262 | 1 |
| C4481 | 2.5 | C4485 | 9 | C4490 | 1.5 | C4498 | 7 |
| C451 | 203 | C4514 | 0.5 | C4518 | 0.5 | C4529 | 1 |
| C809 | 3.5 | C8084 | 1 | C8083 | 5 | C8077 | 1 |
| C4724 | 10 | C4730 | 5.5 | C4732 | 16 | C4737 | 9.5 |
| C4740 | 1 | C4743 | 3 | C4744 | 2.5 | C4746 | 32 |

**Table 6.** Average number $n$ of subsequent users per multi-user computer (continued)
